# Impact of Preoperative Toxicological Screening on Perioperative Anesthetic Management and Short-Term Outcomes Following Metabolic and Bariatric Surgery: A Prospective Observational Study

**DOI:** 10.1007/s11695-026-08534-3

**Published:** 2026-03-31

**Authors:** Mohamed Hany, Adel Ibrahim Hozien, Heba Abdel Samie Mohamed Hussein, Islam El-Sayes, Engi Yousry Hashem, Ehab Elmongui, Ahmed El Shamarka, Walid El Ansari

**Affiliations:** 1 Department of Surgery, Medical Research Institute, Alexandria, Egypt; 2 Bariatric Surgery at Madina Women’s Hospital, Alexandria, Egypt; 3 Assistant Professor of Anaesthesia and Pain Management, Medical Research Institute, Alexandria, Egypt; 4 Assistant Professor of Forensic Medicine and Clinical Toxicology, Faculty of Medicine, Alexandria University, Alexandria, Egypt; 5 Assistant Professor of Surgery, Faculty of Medicine, Alexandria University, Alexandria, Egypt; 6 Lecturer of Anaesthesia and Pain Management, Medical Research Institute, Alexandria University, Alexandria, Egypt; 7 Independent biostatistical consultant, Alexandria, Egypt; 8 Alexandria University, Alexandria, Egypt; 9 The Research Papyrus Lab, Alexandria, Egypt; 10 College of Medicine, Ajman University, Ajman, United Arab Emirates

**Keywords:** Substance use disorder (SUD), Anesthetic management, Metabolic and bariatric surgery, Urine toxicology screening, Substance use, Total weight loss

## Abstract

**Introduction:**

Preoperative substance use is increasingly observed among candidates for metabolic and bariatric surgery (MBS), yet its perioperative and 1-year effects remain unclear in the Middle East and North Africa (MENA). We assessed whether routine preoperative urine toxicology screening tests (TSTs) predict anesthetic requirements, recovery, early complications, and 1-year weight loss.

**Methods:**

Prospective single-center cohort of 1,260 primary MBS patients (Alexandria, Egypt; Dec 2023–May 2024). All underwent urine screening for seven substance classes and were classified as TST-positive or TST-negative. Outcomes included propofol and intraoperative fentanyl doses, recovery metrics, postoperative pain (VAS) and opioid requirements, 30-day complications/readmissions, length of stay, and 1-year percent total and excess weight loss (%TWL, %EWL). Inverse propensity score weighting (IPSW) balanced baseline covariates. One-year outcomes were available for 1,134 of 1,260 patients (90.0%).

**Results:**

190 out of 1,260 (15.1%) were TST-positive—most commonly tramadol (57.9%) and cannabis (36.8%); 12.1% denied use despite a positive TST. After IPSW adjustment, TST-positive patients required more propofol (mean difference 49.7 mg; *p* < 0.001) and fentanyl (MD 51.2 µg; *p* < 0.001), experienced longer recovery times (MD 5.36 min; *p* < 0.001), had higher postoperative VAS scores and opioid requirements, longer hospital stays (*p* < 0.001), and increased 30-day complications (5.4% vs. 1.9%; *p* = 0.015) and readmissions (7.1% vs. 1.6%; *p* < 0.001).

**Conclusions:**

Routine preoperative TST identifies patients at risk for increased anesthetic/analgesic needs, delayed recovery, agitation, and higher early morbidity. Incorporating TST into preoperative pathways may optimize perioperative planning in MENA settings.

**Trial Registration:**

NCT07100314.

**Graphical Abstract:**

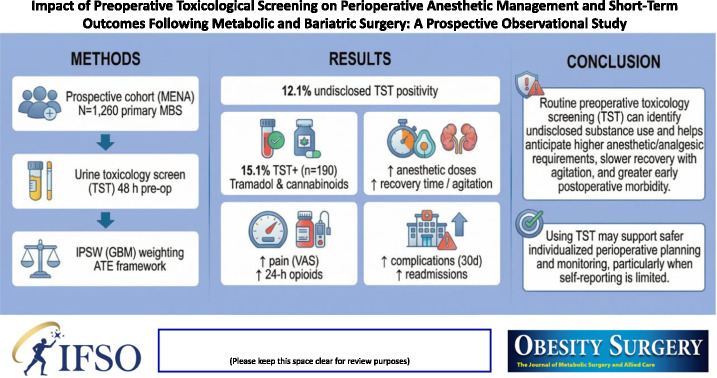

**Supplementary Information:**

The online version contains supplementary material available at 10.1007/s11695-026-08534-3.

## Key Points

TST-positive patients required higher anesthetic doses and longer, agitated recovery.

TST-positive patients reported higher pain scores and greater 24-h opioid consumption.

TST-positive patients had more 30-day chest infections and readmissions for vomiting.

Undisclosed TST-positivity (12.1%) supports objective screening in the MENA region.

**Supplementary Information:**

The online version contains supplementary material available at 10.1007/s11695-026-08534-3.

## Introduction

Obesity is a global health crisis. In 2021, an estimated 1·00 billion adult males and 1·11 billion adult females were overweight and or with obesity. The highest age-standardized prevalence (more than 80% in adults) of overweight and obesity was observed in countries in Oceania, North Africa, and the Middle East [1]. This epidemic has a major influence on public health and healthcare systems, as obesity is associated with numerous health complications, including type 2 diabetes, cardiovascular disease, and reduced quality of life [2, 3].

In the Middle East and North Africa (MENA) region, obesity rates have surged due to urbanization, economic growth, Western dietary influences, and decreased physical activity [1, 4]. Metabolic bariatric surgery (MBS), notably sleeve gastrectomy (SG), Roux-en-Y gastric bypass (RYGB), and one-anastomosis gastric bypass (OAGB), has become the standard intervention for severe obesity, yielding substantial weight loss and remission of obesity-related diseases [5–7].

Recent Substance use disorder (SUD) complicates MBS, affecting anesthesia, recovery, and surgical outcomes, with increased risks among patients misusing opioids, cocaine, or other stimulants [8–10]. These individuals often exhibit altered drug metabolism and exaggerated pain responses requiring higher opioid doses, predisposing them to respiratory depression or withdrawal. Stimulant use may trigger arrhythmia, hypertension, or myocardial ischemia. A multidisciplinary perioperative strategy, integrating addiction counseling, appropriate patient selection, individualized anesthetic planning, multimodal analgesia, and extended monitoring, is essential to optimize the safety and success of MBS [9, 11, 12].

SUD disclosure poses unique challenges in the MENA region due to cultural, religious, and legal constraints, often leading to underreporting during clinical assessment [13]. Regional patterns also differ and higher than the global trends, with higher use of tramadol and cannabis, persistent tobacco consumption, and traditional substances such as khat [12, 14, 15].

Given these disclosure barriers, routine urine toxicology screening holds value in MBS pathways within the MENA region [16]. Notably, Chao et al. found that unexpected toxicology positivity is common among MBS candidates and may influence anesthetic management, though the impact of specific substances on perioperative outcomes remains unclear [17].

Existing studies have not clarified how region-specific substance use patterns affect anesthesia, postoperative emergence, and MBS outcomes. This prospective study examines the prevalence and patterns of recent substance exposure, patient disclosure concordance, intraoperative anesthetic dosing, recovery, postoperative pain, and whether recent substance use leads to higher short-term complications and poorer outcomes in weight loss and obesity-related diseases after MBS. By addressing these gaps, the investigation aims to assess whether routine toxicology screening can enhance surgical and anesthetic decision-making and improve patient safety in cases where self-reported histories may be inaccurate.

## Methods

### Study Design and Setting

The study was conducted in accordance with the Declaration of Helsinki and was reviewed and approved by the Ethics Committee of the medical research institution, Alexandria University (IORG0008812, IRB00010526, Serial Number: E/C. S/N. R28/2023), and the ethical committee of the faculty of medicine at Alexandria University (IRB00012098; FWA NO: 00018699; Serial Number: 0306158) in May 2023. It was also registered in the Clinical Trials Registry (NCT07100314).

All participants provided written informed consent after receiving complete information about the study. Patient confidentiality was maintained throughout the research. The protocol included provisions for participants to withdraw consent at any time without affecting their clinical care. This prospective observational study was conducted from December 2023 to May 2024 at the Medical Research Institute, Alexandria University, Egypt.

### Patient Population

The study involved adult patients (≥ 18 years) with obesity who were scheduled for primary MBS, specifically SG, RYGB, and OAGB. Patient selection was based on the 2022 criteria established by the American Society for Metabolic and Bariatric Surgery (ASMBS) and the International Federation for the Surgery and Other Therapies for Obesity (IFSO) for MBS [18]. The exclusion criteria included patients who had significant abdominal surgeries, emergency procedures, chronic pain conditions, severe psychological issues, patients with dependence on specific substances, such as cocaine and amphetamine, patients who refused participation, did not adhere to study protocols, or could not complete follow-up evaluations.

### Preoperative Workup

#### History, Physical Examination, and Preoperative Education

A thorough and detailed medical and surgical history was obtained, including medication use and history of SUD. Special attention was given to self-reported use of tobacco, alcohol, prescription medications, and illegal drugs. The physical exam included an airway assessment, standard measurements (height, weight, and BMI), and a comprehensive systems review to identify potential surgical risks. All patients participated in a structured education program that covered anesthesia, surgery, expected outcomes, potential complications, and necessary lifestyle adjustments.

#### Laboratory Investigations

Standard preoperative laboratory evaluation (Table 1). Additional tests such as echocardiography, stress testing, and pulmonary function tests were ordered based on individual patient factors and associated medical issues. Routine evaluations also included Esophagogastroduodenoscopy (EGD) and ultrasound examination of the abdomen and pelvis.

**Table 1:** Comparison of demographic, clinical, and laboratory characteristics between TST-positive and TST-negative participants undergoing metabolic and bariatric surgery (*N* = 1,260). Data are presented as mean ± standard error (SE) or n (%). Comparisons were performed using independent sample t-tests for continuous variables and Chi-square or fisher’s exact tests for categorical variables, as appropriate. Statistically significant differences (*p* < 0.05) are highlighted in bold italic

Variable	Total (*N* = 1260)	TST Positive (*n* = 190)	TST Negative (*n* = 1070)	*p*
Age	36.8 ± 0.3	37.5 ± 0.8	36.7 ± 0.3	0.394
Sex				
Female	891 (70.7)	126 (66.3)	765 (71.5)	0.166
Male	369 (29.3)	64 (33.7)	305 (28.5)	
Anthropometrics				
Height	166.1 ± 0.3	166.1 ± 0.7	166.1 ± 0.3	0.977
Weight	124.7 ± 0.7	123.8 ± 1.8	124.8 ± 0.8	0.625
BMI	45.1 ± 0.2	44.7 ± 0.5	45.2 ± 0.2	0.383
Type of MBS				
SG	1170 (92.9)	159 (83.7)	1011 (94.5)	***< 0.001***
OAGB	19 (1.5)	11 (5.8)	8 (0.7)	***0.003***
RYGB	71 (5.6)	20 (10.5)	51 (4.8)	***< 0.001***
Operative time (min)	47.5 ± 0.1	47.6 ± 0.3	47.5 ± 0.1	0.800
Smoking	245 (19.4)	65 (34.2)	180 (16.8)	***< 0.001***
Self-Reported substance use				
No	1093 (87.1)	23 (12.1)	1070 (100.0)	***< 0.001***
Yes	167 (13.3)	167 (87.9)	0 (0.0)	
Obesity-related diseases				
OSA	623 (49.4)	92 (48.4)	531 (49.6)	0.813
Hypertension	267 (21.2)	47 (24.7)	220 (20.6)	0.210
Osteoarthritis	259 (20.6)	46 (24.2)	213 (19.9)	0.174
Diabetes	153 (12.1)	26 (13.7)	127 (11.9)	0.471
Dyslipidemia	128 (10.2)	23 (12.1)	105 (9.8)	0.361
Insulin resistance	107 (8.5)	13 (6.8)	94 (8.8)	0.480
Lab Investigations				
HGB (g/dL)	12.9 ± 0.0	12.9 ± 0.1	12.9 ± 0.0	0.842
PLT (×10⁹/L)	295.2 ± 2.1	295.8 ± 5.3	295.0 ± 2.3	0.893
WBC(×10⁹/L)	8.8 ± 0.5	7.7 ± 0.2	9.0 ± 0.6	0.024
Clotting time (Seconds)	434.4 ± 3.9	431.1 ± 9.1	435.0 ± 4.3	0.700
Bleeding time (Seconds)	146.6 ± 2.4	137.9 ± 5.9	148.2 ± 2.6	0.112
Prothrombin (Seconds)	14.9 ± 1.7	11.9 ± 0.1	15.5 ± 1.9	0.068
Prothrombin Activity (%)	94.2 ± 0.3	95.5 ± 0.7	94.0 ± 0.3	***0.033***
PTT (Seconds)	30.9 ± 0.1	31.0 ± 0.3	30.9 ± 0.1	0.955
INR	1.0 ± 0.0	1.0 ± 0.0	1.0 ± 0.0	0.946
Urea (mg/dL)	26.8 ± 0.2	26.8 ± 0.6	26.9 ± 0.2	0.910
Creatinine (mg/dL)	0.8 ± 0.0	0.8 ± 0.0	0.8 ± 0.0	0.385
SGOT (U/L)	22.2 ± 0.4	22.2 ± 0.8	22.2 ± 0.5	0.984
SGPT (U/L)	24.1 ± 0.4	24.4 ± 0.9	24.1 ± 0.4	0.724
FBS (mg/dL)	98.8 ± 0.8	98.7 ± 2.1	98.9 ± 0.8	0.927
HBA1C (%)	6.0 ± 0.3	5.7 ± 0.1	6.1 ± 0.4	0.313
Triglycerides (mg/dL)	135.3 ± 1.5	138.2 ± 4.2	134.8 ± 1.7	0.455
Cholesterol (mg/dL)	169.9 ± 1.2	171.4 ± 3.3	169.6 ± 1.3	0.617
TSH (µIU/mL)	2.1 ± 0.0	2.1 ± 0.1	2.1 ± 0.0	0.676
FT3 (pg/mL)	3.4 ± 0.3	3.1 ± 0.1	3.4 ± 0.4	0.488
FT4 (ng/dL)	1.3 ± 0.0	1.3 ± 0.1	1.3 ± 0.0	0.779
HBV positive	7 (0.6)	2 (1.1)	5 (0.5)	0.286
HBC positive	12 (1.0)	0 (0.0)	12 (1.1)	0.232

Continuous variables are presented as mean ± standard error (SE), and categorical variables as number and percentage n (%). TST, toxicology screening test; MBS, metabolic and bariatric surgery; SG, sleeve gastrectomy; OAGB, one-anastomosis gastric bypass; RYGB, Roux-en-Y gastric bypass; OSA, obstructive sleep apnea; COPD, chronic obstructive pulmonary disease; DVT, deep vein thrombosis; HGB, hemoglobin; PLT, platelet count; WBC, white blood cell count; SGOT, serum glutamic-oxaloacetic transaminase; SGPT, serum glutamic-pyruvic transaminase; FBS, fasting blood sugar; HbA1c, glycated hemoglobin A1c; TSH, thyroid-stimulating hormone; FT3, free triiodothyronine; FT4, free thyroxine; HBV, hepatitis B virus; HCV, hepatitis C virus; BMI, body mass index. P values were calculated using independent sample t-tests for continuous variables and Chi-square or Fisher’s exact tests for categorical variables, as appropriate. Statistically significant values (*p* < 0.05) are highlighted in bold italics

#### Toxicological Screening

All participants underwent urine toxicology screening 48 h before surgery using a standardized multi-drug immunoassay dipstick panel (Assure Tech DOA Dipstick Screen Panel, model MD-U512; Assure Tech, Hangzhou, China). This point-of-care test enables rapid qualitative detection. The panel screened for seven substance groups that are most commonly misused in MENA region and relevant to perioperative anesthetic management: cocaine metabolites (COC), indicating recent cocaine exposure; amphetamines (AMP); cannabis metabolite detected as tetrahydrocannabinol (THC); morphine and related opiate compounds (MOP) such as codeine; tramadol (TRA); barbiturates (BAR); and benzodiazepines (BZO). Each target represented a category of substances with known potential to alter anesthesia pharmacodynamics, postoperative analgesic requirements, or recovery physiology.

Screening procedures followed established clinical and forensic protocols, including the supervised collection of at least 30 ml of urine to ensure sample integrity [19, 20]. Results were interpreted according to manufacturer specifications: a visible red-to-pink line at the test region (T) indicated a negative result, while its absence indicated a positive detection. Both the surgeon and anesthetist were blinded to the TST results, which were accessible only to the research team and not communicated to the clinical or data collection teams until after the patient’s discharge from the PACU.

#### Smoking

Active tobacco use was assessed in all participants during preoperative evaluation because of its potential impact on anesthesia and postoperative respiratory risk [21, 22]. Patients who are currently smoking received counseling encouraging them to stop smoking for 4–6 weeks before surgery [23].

#### Nutritional, Psychological, and Subspecialty Consultations

A dietitian performed a nutritional assessment and provided preoperative dietary education, while a mental health professional evaluated patients’ psychological readiness for surgery and risk for substance misuse. Customized consultations were also conducted in endocrinology, pulmonology, and cardiology.

#### Anesthetic Management and Analgesic Protocol

All patients underwent a standardized anesthetic protocol with ASA Standard monitoring. Following pre-oxygenation in the RAMP position, induction included fentanyl (1 µg/kg), a titrated dose of propofol until loss of verbal response, and rocuronium (0.6 mg/kg) for intubation. Maintenance with sevoflurane (MAC 2%) and Dexmedetomidine (0.2–0.5 mcg/kg/hr), with mechanical ventilation set to 6–8 ml/kg tidal volume, 12–15 breath/min, and a PEEP of 8–10 cmH₂O to keep end-tidal CO2 at 35–40 mmHg. Prior to incision, bilateral TAP blocks were performed with 20 mL of 0.2% bupivacaine per side, augmented with local anesthetic infiltration at the trocar site. Incremental rocuronium doses were administered as needed. Additional fentanyl (0.25 mcg/kg) was given if heart rate or systolic blood pressure increased by > 20% from baseline. Intraoperative medications included dexamethasone (8 mg), granisetron (1 mg), pantoprazole (40 mg), paracetamol (1 g), and ketorolac (30 mg). At the end of surgery, the inhalational anesthetic and dexmedetomidine were discontinued, and neuromuscular blockade was reversed with sugammadex (2 mg/kg) followed by extubation in a semi-sitting position. Postoperatively, all patients received multimodal analgesia in the form of intravenous paracetamol (1 g/6 h) and ketorolac (30 mg/8 h), and a PCA pump was connected for self-administered 2 mg morphine if the VAS ≥ 4 with a 15-minute lockout and max 20 mg over 4 h. Granisetron (1 mg IV/day) was given as an antiemetic strategy with good hydration.

### Data Collection and Variables

Baseline variables, including age, sex, BMI, socioeconomic status, and medical history (with attention to chronic pain and smoking), were collected for all participants. A substance misuse history was obtained through structured interviews conducted by trained staff, distinguishing between prescribed and non-prescribed exposure, and only patients with SUD were included.

Anesthetic outcomes, including total propofol dose, intraoperative fentanyl requirements, and duration of surgery. Recovery time was measured from extubation to a modified Aldrete score ≥ 9 [24], and recovery status was assessed using the Richmond Agitation–Sedation Scale (RASS) (− 5 to + 4, with 0 indicating calm alertness) [25]. Postoperative pain was evaluated via the Visual Analog Scale (VAS, 0–10) at 1, 6, 12, and 24 h, total morphine consumption, and the time to first opioid request were documented.

Short-term surgical outcomes included the incidence of 30-day complications and were categorized according to standardized MBS reporting criteria [26], as well as hospital length of stay and 30-day readmission rates. One-year post-surgery outcomes were assessed using absolute weight change, BMI reduction, percentage total weight loss (%TWL), and percentage excess weight loss (%EWL). Only patients with available one-year follow-up data were included in these analyses. Resolution or improvement of obesity-related medical problems, including type 2 diabetes, hypertension, and dyslipidemia, was determined based on standardized metabolic outcome definitions in MBS [26].

### Sample Size Calculation and Statistical Analysis

The study aimed to determine the sample size necessary to assess the prevalence of substance use disorder (SUD) among patients undergoing bariatric metabolic surgery (BMS), setting a 95% confidence level and a 3% margin of error. Using a conservative prevalence estimate (p = 0.5), the study calculated a required sample size of 1,068 participants via the ‘epi.sssimpleestb’ function in R, ultimately recruiting 1,260 participants to allow an 18% safety margin. Statistical analyses employed R version 4.4.2, applying descriptive statistics to summarize baseline characteristics, independent t-tests for continuous variables, and Chi-square or Fisher’s exact tests for categorical variables. The reliability of self-reported SUD was assessed with Cohen’s Kappa statistics.

To manage confounding variables, Inverse Propensity Score Weighting (IPSW) was employed using the TWANG package, implementing a Gradient Boosting Machine (GBM) model with covariates such as age, sex, type of MBS, preoperative BMI, and various comorbidities. The optimal number of GBM trees was determined to be 1,750, with balance improvement confirmed through aSMD evaluations. Post-weighting, effective sample sizes were reported as 158.5 for the TST-positive group and 1,053.1 for the TST-negative group, enhancing balance but slightly undermining precision, particularly in the TST-positive group.

The analysis addressed missing data via Little’s MCAR test, concluding that the missing data (≤ 7%) adhered to MCAR assumptions. Generalized Estimating Equations (GEE) were utilized to analyze longitudinal changes over one year and postoperative pain scores, accommodating repeated measures and missing data while maintaining unbiased estimates. Statistical significance was set at a two-tailed p-value < 0.05 for all analyses.

## Results

**Fig. 1:**
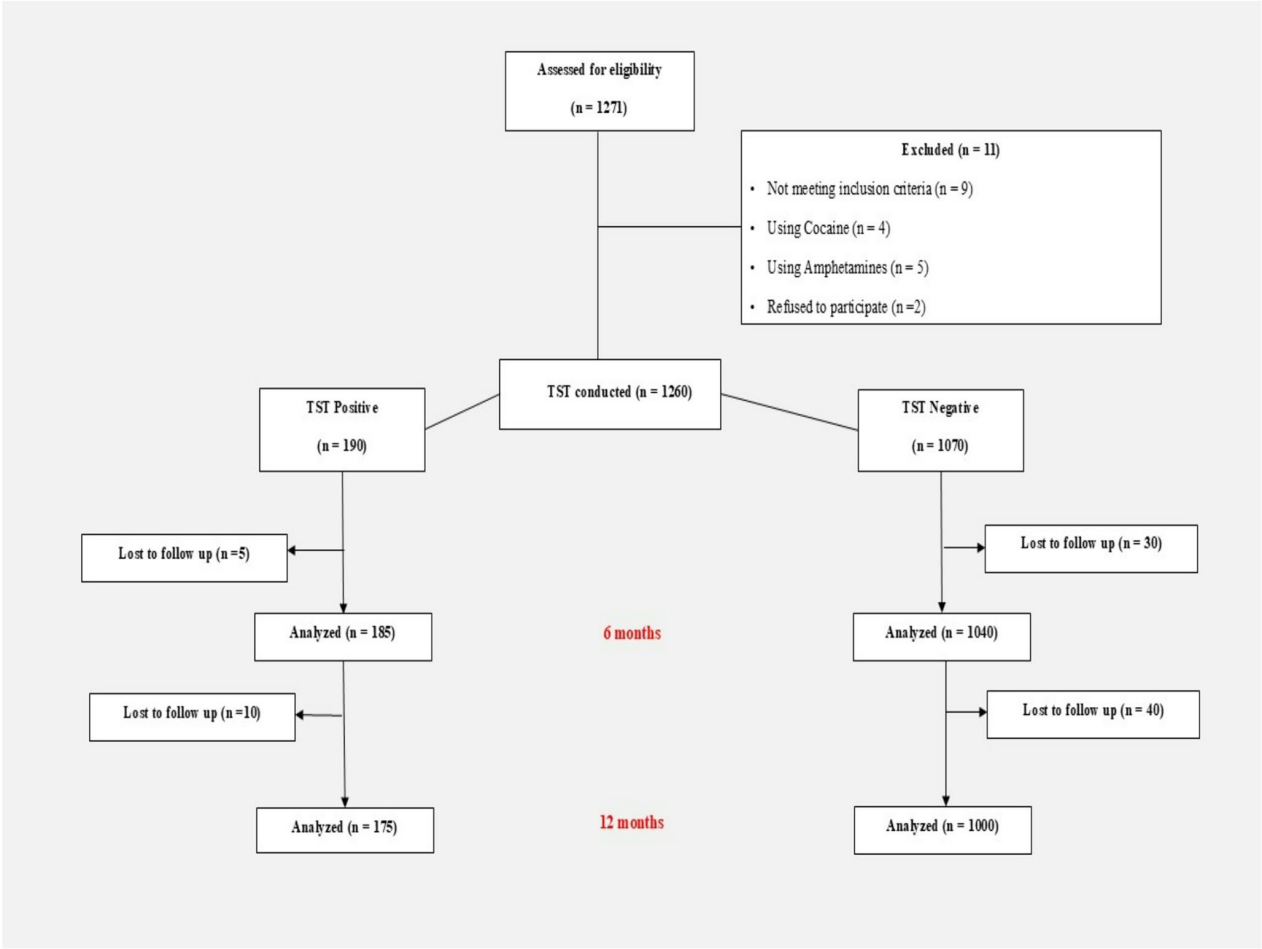
Participant flowchart showing recruitment and follow-up

**Fig. 2:**
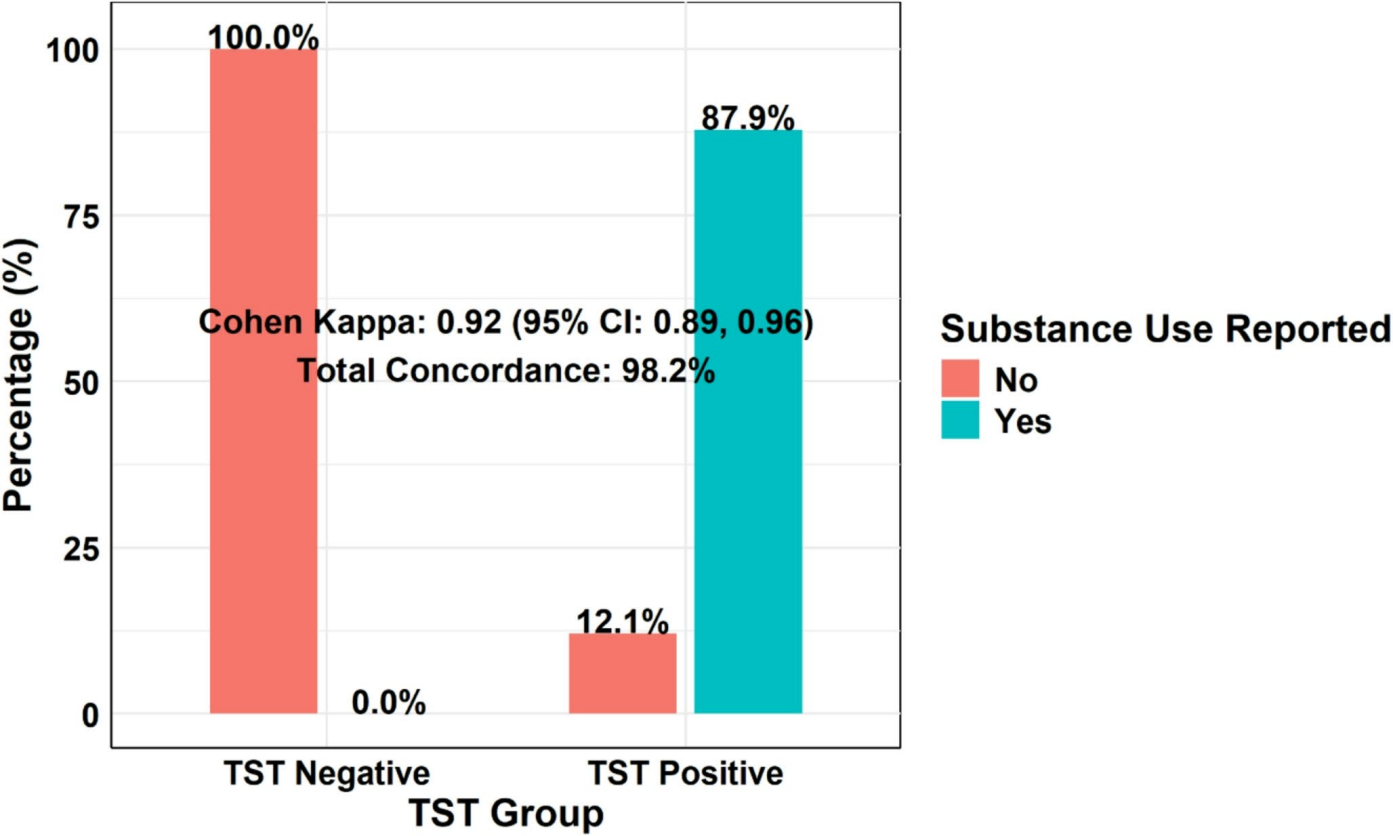
Agreement between self-reported substance use and toxicology screening test (TST) results. Bar graph showing the percentage of self-reported substance use among TST-positive and TST-negative participants. All TST-negative participants reported no substance use, whereas 87.9% of TST-positive participants reported substance use. Agreement between TST results and self-reported data was assessed using Cohen’s kappa, demonstrating very high concordance (κ = 0.92; 95% CI: 0.89–0.96), with an overall agreement rate of 98.2%

The (Fig. 1) illustrates the flowchart of study participants. Of the 1,271 individuals assessed for eligibility, 11 were excluded: 9 for not meeting inclusion criteria (including 4 using cocaine and 5 using amphetamines) and two who refused to participate. The remaining 1,260 participants underwent the TST. The 6-month follow-up showed the loss of 5 TST-positive and 30 TST-negative participants. By the 1-year mark, an additional 10 TST-positive and 40 TST-negative participants were lost. All available data at each time point were included in the analyses. Self-reported substance use (Fig. 2) showed strong agreement with TST (Cohen’s κ = 0.92; 95% CI, 0.89–0.96), although 12.1% of positive cases were unreported.

### Study Population and Baseline Characteristics

The study population had a mean age of 36.8 ± 0.3 years, with the majority of patients being female (*n* = 891, 70.7%). Of the 1,260 patients, 190 (15.1%) tested positive on preoperative TST. The mean preoperative BMI was 45.1 ± 0.2 kg/m². The same surgery team performed all the surgeries, and the duration of surgery was comparable between the two groups (Table 1). SG was the most common procedure, performed in 1,170 patients (92.9%), followed by RYGB in 71 patients (5.6%), and OAGB in 19 patients (1.5%). Smoking was reported in 245 patients (19.4%) overall, and in 34.2% of TST-positive participants compared to 16.8% of TST-negative participants (*p* < 0.001) (Table 1). After IPSW, all baseline characteristics were well balanced between groups (Table 2).

**Table 2:** Operative characteristics, anesthesia requirements, patient-reported pain scores, postoperative morphine requirements, and hospital length of stay comparing TST-positive and TST-negative participants. Data are presented as mean ± standard error (SE). Mean differences (MD) are shown with 95% confidence intervals (CI). IPSW weighting using a generalized boosted model was applied to balance baseline covariates under the average treatment effect (ATE) framework; weighted sample sizes reflect pseudo-populations, and effective sample sizes (ESS) are provided to account for variance inflation. Linear regression models were used to compare anesthesia requirements, opioid consumption, and length of stay in both unadjusted and weighted analyses, while repeated VAS measurements were analyzed using generalized estimating equations (GEE). Statistically significant differences (*p* < 0.05) are shown in bold italic

Operative data	Unadjusted Analysis				IPSW Adjusted Analysis			
	TST Positive (*n* = 190)	TST Negative (*n* = 1070)	MD (95% CI)	*p*	TST Positive (*n* = 945)	TST Negative (*n* = 1248)	MD (95% CI)	*p*
Anesthesia requirements								
Propofol dose mg	214.9 ± 1.7	166.7 ± 0.4	48.24 (44.75, 51.72)	***< 0.001***	216.5 ± 1.8	166.7 ± 0.4	49.67 (46.17, 53.16)	***< 0.001***
Peri-operative fentanyl dose (mcg	174.5 ± 1.2	124.7 ± 0.5	49.80 (47.28, 52.32)	***< 0.001***	175.8 ± 1.2	124.6 ± 0.5	51.18 (48.63, 53.73)	***< 0.001***
Recovery time (min)	15.1 ± 0.2	10.0 ± 0.1	5.12 (4.60, 5.64)	***< 0.001***	15.4 ± 0.2	10.0 ± 0.1	5.36 (4.84, 5.87)	***< 0.001***
Recovery status score	1.9 ± 0.1	−0.5 ± 0.0	2.43 (2.26, 2.60)	***< 0.001***	2.0 ± 0.1	−0.5 ± 0.0	2.48 (2.30, 2.66)	***< 0.001***
Patient-reported pain scores (VAS)								
Baseline	1.6 ± 0.0	0.6 ± 0.0	0.99 (0.89, 1.09)	***< 0.001***	1.6 ± 0.1	0.6 ± 0.0	1.01 (0.90, 1.12)	***< 0.001***
One hour	4.9 ± 0.1	3.8 ± 0.0	1.03 (0.90, 1.16)	***< 0.001***	4.9 ± 0.1	3.8 ± 0.0	1.05 (0.91, 1.19)	***< 0.001***
6 h	3.9 ± 0.1	2.8 ± 0.0	1.08 (0.95, 1.21)	***< 0.001***	3.9 ± 0.1	2.8 ± 0.0	1.10 (0.96, 1.24)	***< 0.001***
12 h	2.9 ± 0.1	2.1 ± 0.0	0.80 (0.67, 0.94)	***< 0.001***	2.9 ± 0.1	2.0 ± 0.0	0.84 (0.70, 0.99)	***< 0.001***
24 h	2.8 ± 0.1	0.8 ± 0.0	1.98 (1.86, 2.10)	***< 0.001***	2.8 ± 0.1	0.8 ± 0.0	2.00 (1.88, 2.13)	***< 0.001***
First opioid request if VAS ≥ 4 (hour)	2.5 ± 0.1	3.6 ± 0.0	−1.08 (−1.27, −0.89)	***< 0.001***	2.6 ± 0.1	3.6 ± 0.0	−0.96 (−1.16, −0.77)	***< 0.001***
Post-operative morphine dose (mg)	6.2 ± 0.1	2.7 ± 0.1	3.46 (3.16, 3.75)	***< 0.001***	6.2 ± 0.1	2.7 ± 0.1	3.55 (3.25, 3.85)	***< 0.001***
Hospital stay	2.1 ± 0.0	2.0 ± 0.0	0.12 (0.05, 0.18)	***< 0.001***	2.1 ± 0.0	2.0 ± 0.0	0.12 (0.05, 0.19)	***< 0.001***

Continuous variables are reported as mean ± standard error (SE). TST: Toxicology Screening Test; VAS: Visual Analog Scale. MD: Mean Difference with 95% Confidence Interval (CI). IPSW adjustment was performed using a generalized boosted model to balance baseline covariates under the ATE framework. Sample sizes shown in the adjusted columns reflect inflated totals due to weighting and do not represent the actual number of participants. Effective sample sizes (ESS), which account for variance inflation, were 158.5 for TST-positive and 1,053.1 for TST-negative groups. p-values for anesthesia requirements, morphine use, and hospital stay are based on linear regression models before and after weighting. p-values for VAS pain scores were derived from Generalized Estimating Equations (GEE) accounting for repeated measures over time. Statistically significant p-values (*p* < 0.05) are shown in bold italic

**Fig. 3:**
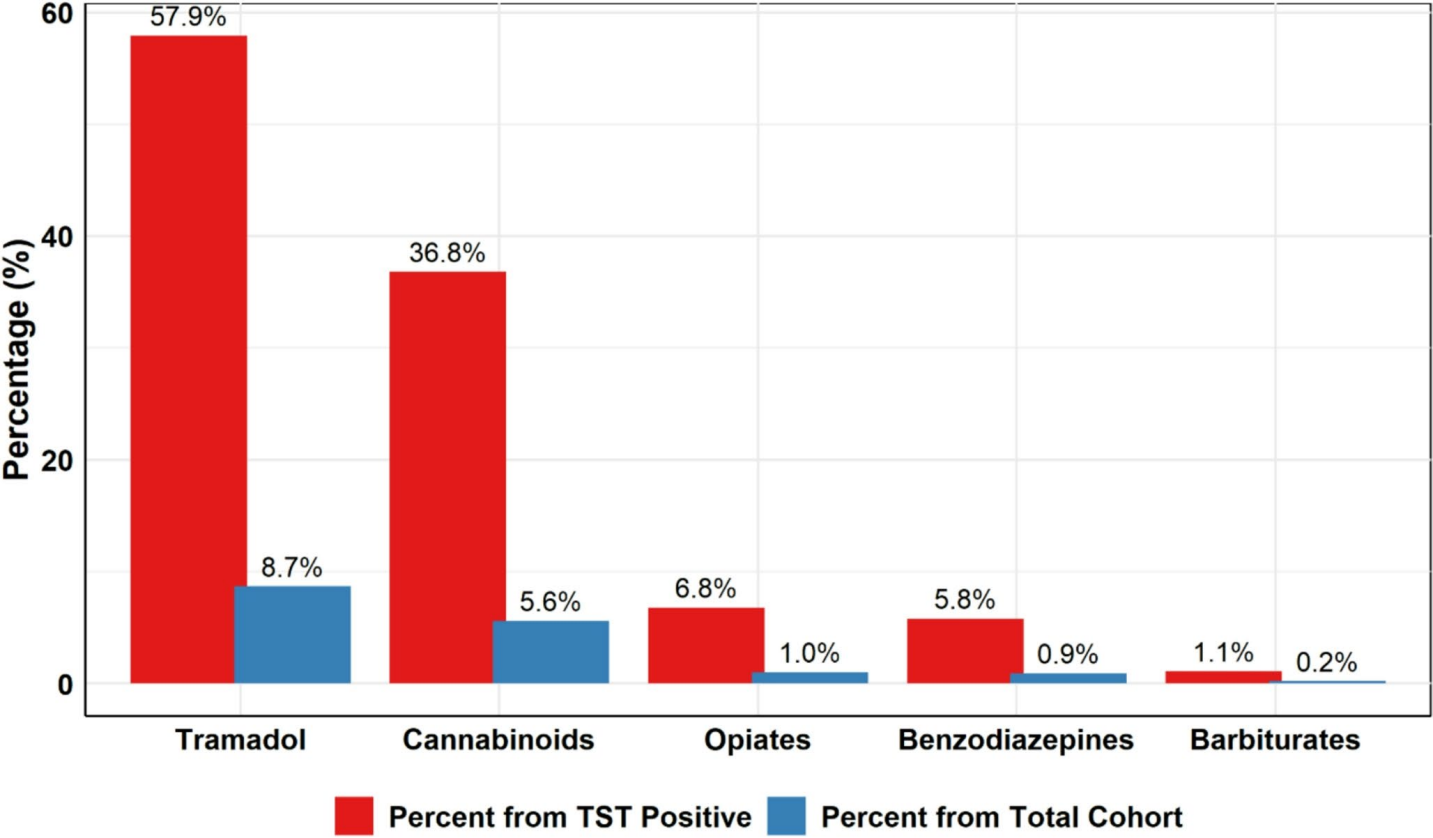
Distribution of detected substances among TST-positive patients and in the overall study cohort. Bar chart illustrating the prevalence of individual substances detected by toxicology screening among TST-positive patients, compared with their prevalence in the entire study population. Tramadol and cannabinoids were the most frequently detected substances among TST-positive cases. Opiates, benzodiazepines, and barbiturates were detected at lower frequencies

Multiple substances were detected (Fig. 3), tramadol was found in 57.9% of TST-positive cases and 8.7% of the overall cohort. Cannabinoids were present in 36.8% of TST-positive individuals and 5.6% of the entire cohort. Other detected substances include opiates, benzodiazepines and barbiturates.

### Anesthesia Requirements and Perioperative Outcomes. (Table 2)

The TST-positive patients required significantly higher anesthetic doses. The mean propofol induction dose was 214.9 ± 1.7 mg in the TST-positive group versus 166.7 ± 0.4 mg in the TST-negative group (*p* < 0.001). Similarly, intraoperative fentanyl requirements were substantially higher in TST-positive patients (174.5 ± 1.2 mcg vs. 124.7 ± 0.5 mcg, *p* < 0.001). Recovery time was significantly longer in the TST-positive group (15.1 ± 0.2 vs. 10.0 ± 0.1 min, *p* < 0.001). Recovery status scores were higher in the TST-positive group (1.9 ± 0.1 vs. −0.5 ± 0.0, *p* < 0.001).

After IPSW adjustment, the TST-positive patients still required higher mean doses of propofol (49.67 mg, 95% CI: 46.17–53.16) and fentanyl (51.18 mcg, 95% CI: 48.63–53.73) (*p* < 0.001). Recovery time was longer (MD = 5.36 min, 95% CI: 4.84–5.87), and recovery status scores were higher (MD = 2.48, 95% CI: 2.30–2.66) (*p* < 0.001).

The VAS was consistently higher in TST-positive patients at all time points during the first 24 h after surgery (*p* < 0.001). The mean VAS scores peaked at 1 h postoperatively in both groups, but the TST-positive group reported higher scores at all assessments (baseline, 1, 6, 12, and 24 h). The time to first opioid request was shorter in the TST-positive group (2.5 ± 0.1 vs. 3.6 ± 0.0, *p* < 0.001), and postoperative morphine consumption was higher (6.2 ± 0.1 vs. 2.7 ± 0.1, *p* < 0.001). A statistically significant difference in hospital stay duration was also observed in the TST-positive group (2.1 ± 0.0 vs. 2.0 ± 0.0, MD = 0.12 days, 95% CI: 0.05–0.18, *p* < 0.001).

**Fig. 4:**
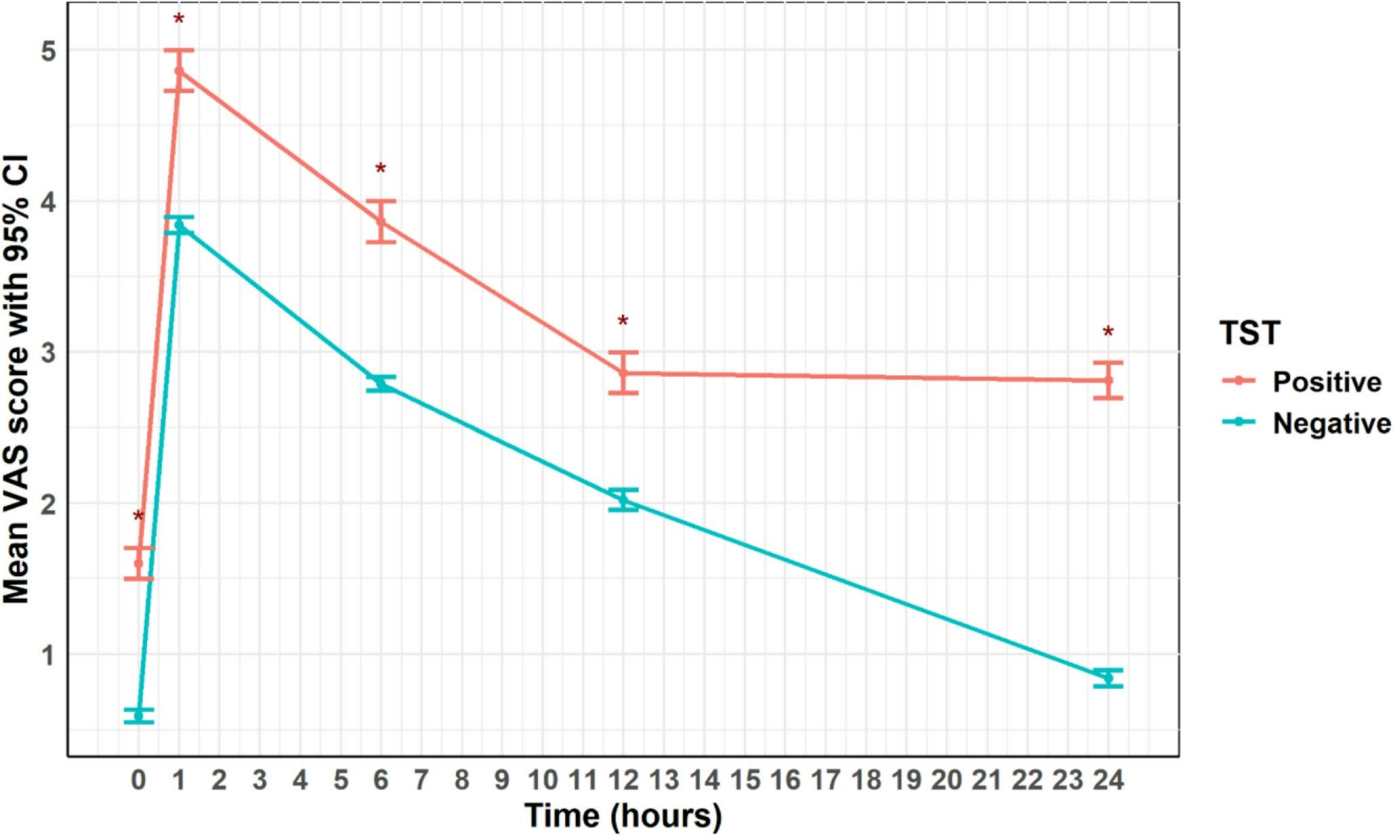
Inverse propensity score–weighted postoperative visual analogue scale (VAS) pain scores over 24 h by TST group. Inverse propensity score–weighted (IPSW) mean Visual Analog Scale (VAS) pain scores with 95% confidence intervals at five postoperative time points (baseline, 1 h, 6 h, 12 h, and 24 h), stratified by TST-positive and TST-negative groups. Repeated measurements were analyzed using generalized estimating equations (GEE) to account for within-patient correlation. Across all time points, the TST-positive group reported higher pain scores. Asterisks (*) indicate statistically significant between-group differences (*p* < 0.05). Unadjusted results are presented in Supplementary Figure S4

GEE analysis confirmed that TST-positive status was independently associated with higher pain scores (Fig. 4) and morphine consumption during the first 24 h (MD = 3.55 mg, 95% CI: 3.25, 3.85, *p* < 0.001). The time to first analgesic request was also shorter in the TST-positive group (MD = −0.96 min, 95% CI: −1.16 to −0.77, *p* < 0.001), suggesting an earlier onset of significant postoperative pain.

### Short-Term Complications and Readmissions. (Table 3)

**Table 3:** Postoperative complications and readmissions in TST-positive versus TST-negative participants, presented as frequency and percentage of events in both unadjusted and IPSW-adjusted analyses. Between-group comparisons were conducted using logistic regression models before and after weighting to evaluate the association between toxicology screening status and adverse postoperative outcomes. Statistically significant differences (*p* < 0.05) are shown in bold italic

Variable	Total	Unadjusted Analysis			IPSW adjusted Analysis		
		TST Positive (*n* = 190)	TST Negative (*n* = 1070)	*p*	TST Positive (*n* = 945)	TST Negative (*n* = 1248)	*p*
Postoperative Complications	28 (2.2)	9 (4.7)	19 (1.8)	***0.027***	51 (5.4)	24 (1.9)	***0.015***
Type of Postoperative complications:							
Bleeding	7 (0.6)	2 (1.1)	5 (0.5)	0.286	8 (0.9)	6 (0.5)	0.497
Chest Infection	8 (0.6)	4 (2.1)	4 (0.4)	***0.021***	28 (3.0)	5 (0.4)	***< 0.001***
Internal Hernia	1 (0.1)	0 (0.0)	1 (0.1)	1.000	0 (0.0)	3 (0.2)	0.383
Port Site Hernia	1 (0.1)	0 (0.0)	1 (0.1)	1.000	0 (0.0)	1 (0.1)	0.385
Superficial Wound Infection	1 (0.1)	1 (0.5)	0 (0.0)	0.151	7 (0.7)	0 (0.0)	0.249
UTI	3 (0.2)	2 (1.1)	1 (0.1)	0.061	8 (0.8)	1 (0.1)	***0.034***
Wound Infection	1 (0.1)	0 (0.0)	1 (0.1)	1.000	0 (0.0)	1 (0.1)	0.385
Readmission	29 (2.3)	14(7.4)	15 (1.4)	***< 0.001***	67 (7.1)	20 (1.6)	***< 0.001***
Cause of readmission:							
Bleeding	7 (0.6)	2 (1.1)	5 (0.5)	0.286	8 (0.9)	6 (0.5)	0.497
Internal Hernia	1 (0.1)	0 (0.0)	1 (0.1)	1.000	0 (0.0)	3 (0.2)	0.383
Nausea	6 (0.5)	6 (3.2)	0 (0.0)	***< 0.001***	29 (3.1)	0 (0.0)	***0.005***
Port Site Hernia	1 (0.1)	0 (0.0)	1 (0.1)	1.000	0 (0.0)	1 (0.1)	0.385
Vomiting	14 (1.1)	6 (3.2)	8 (0.7)	***0.011***	30 (3.2)	10 (0.8)	***0.009***

Cell values represent frequency (%); TST: Toxicology Screening Test; UTI: Urinary Tract Infection. IPSW adjustment was performed using a generalized boosted model to balance baseline covariates under the ATE framework. Sample sizes in the adjusted columns reflect inflated totals due to weighting, not actual counts. Effective sample sizes (ESS) were 158.5 (TST-positive) and 1,053.1 (TST-negative). P-values are based on logistic regression models before and after weighting. Statistically significant p-values (*p* < 0.05) are shown in bold italic

The IPSW incidence of 30-day complications was significantly higher in TST-positive patients compared to TST-negative patients (5.4% vs. 1.9%, *p* = 0.015). Chest infection was the most frequently reported complication with a statistically significant difference (3.0% vs. 0.4%, *p* < 0.001). Other complications, such as bleeding, superficial wound infection, and urinary tract infection (UTI), showed higher rates among TST-positive participants but did not consistently reach statistical significance.

IPSW readmissions were significantly more common among TST-positive participants (7.1% vs. 1.6%, *p* < 0.001). Vomiting and nausea were the leading causes of readmission requiring intravenous fluid administration.

### Weight Loss Outcomes. (Table 4) (Fig. 5).

**Table 4:** Weight loss outcomes over time in TST-positive and TST-negative participants undergoing metabolic and bariatric surgery, presented for both unadjusted and IPSW-adjusted analyses. Continuous data are reported as mean ± standard error (SE), with mean differences (MD) and 95% confidence intervals (CI) reflecting between-group contrasts at each time point. Generalized estimating equations (GEE) were used to evaluate repeated measures of weight-loss outcomes over time. Statistically significant differences (*p* < 0.05) are shown in bold italic

variable	Unadjusted Analysis				IPSW adjusted Analysis			
	TST Positive	TST Negative	MD (95% CI)	*p*	TST Positive	TST Negative	MD (95% CI)	*p*
Weight (Kg)								
Baseline	123.8 ± 1.8	124.8 ± 0.8	−0.97 (−4.86, 2.91)	0.624	125.6 ± 1.9	125.0 ± 0.8	0.44 (−3.47, 4.36)	0.824
Six months	92.5 ± 1.4	94.3 ± 0.6	−1.18 (−4.15, 1.79)	0.437	94.1 ± 1.4	94.3 ± 0.6	−0.25 (−3.22, 2.72)	0.867
Year 1	82.0 ± 1.3	80.5 ± 0.5	1.23 (−1.48, 3.94)	0.372	82.8 ± 1.3	80.4 ± 0.5	2.27 (−0.43, 4.96)	0.099
BMI (Kg/m ^2^)								
Baseline	44.7 ± 0.5	45.2 ± 0.2	−0.50 (−1.64, 0.63)	0.382	45.4 ± 0.6	45.2 ± 0.2	0.18 (−1.02, 1.37)	0.773
Six months	33.6 ± 0.4	34.2 ± 0.2	−0.53 (−1.48, 0.41)	0.268	34.1 ± 0.5	34.1 ± 0.2	−0.06 (−1.05, 0.94)	0.912
Year 1	29.6 ± 0.4	29.2 ± 0.2	0.34 (−0.50, 1.18)	0.427	29.9 ± 0.4	29.1 ± 0.2	0.83 (−0.05, 1.72)	0.066
TWL (%)								
Six months	24.6 ± 0.5	24.3 ± 0.2	0.31 (−0.66, 1.29)	0.527	24.9 ± 0.5	24.4 ± 0.2	0.38 (−0.65, 1.42)	0.468
Year 1	33.9 ± 0.5	35.1 ± 0.2	−1.26 (−2.40, −0.12)	***0.030***	33.8 ± 0.6	35.1 ± 0.2	−1.35 (−2.61, −0.10)	***0.035***
EWL (%)								
Six months	50.0 ± 1.1	48.7 ± 0.5	1.27 (−1.01, 3.56)	0.275	49.6 ± 1.1	49.0 ± 0.5	0.47 (−1.92, 2.86)	0.700
Year 1	68.2 ± 1.2	69.8 ± 0.5	−1.63 (−4.18, 0.92)	0.211	67.1 ± 1.3	69.9 ± 0.5	−2.87 (−5.63, −0.12)	***0.041***

Cell values represent Mean ± standard error; MD: Mean difference; CI: Confidence Interval; TST: Toxicology Screening Test; BMI: Body Mass Index; %EWL: Percent Excess Weight Loss; %TWL: Percent Total Weight Loss. IPSW adjustment was performed using a generalized boosted model to balance baseline covariates under the average treatment effect (ATE) framework. Sample sizes in the adjusted columns reflect inflated totals resulting from weighting and do not represent the actual number of participants. Effective sample sizes were 158.5 for the TST-positive group and 1,053.1 for the TST-negative group. P-values were derived from Generalized Estimating Equations (GEE) for repeated measures. Statistically significant p-values (*p* < 0.05) are shown in bold italic

Follow-up data for one year were available for 1,134 patients (90.0% of the initial cohort), with similar follow-up rates between TST-positive (88.9%) and TST-negative (90.2%) groups (*p* = 0.62). Both groups experienced marked reductions in body weight and BMI over time, with no statistically significant differences.

**Fig. 5:**
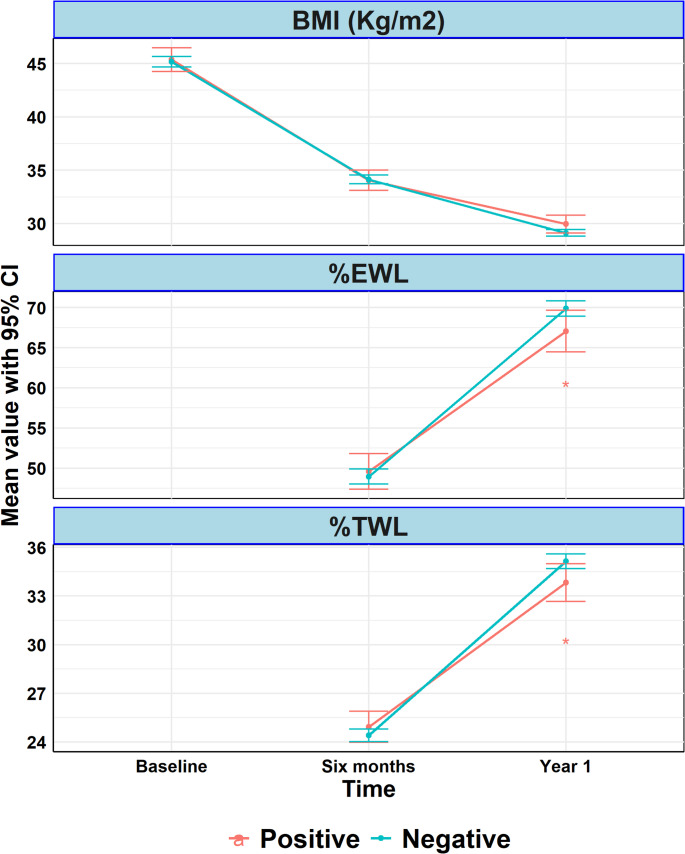
Inverse propensity score–weighted changes in body mass index (BMI), percent excess weight loss (%EWL), and percent total weight loss (%TWL) over one year by TST group. Inverse propensity score–weighted (IPSW) mean values with 95% confidence intervals for body mass index (BMI), percent excess weight loss (%EWL), and percent total weight loss (%TWL) measured at baseline, 6 months, and 1 year after bariatric surgery, stratified by TST group. BMI decreased over time in both groups without significant between-group differences. At 1 year, small differences in %EWL and %TWL were observed between groups. Asterisks (*) denote statistically significant between-group differences (*p* < 0.05). Unadjusted results are shown in Supplementary Figure S5

In the adjusted analysis, %TWL was slightly lower in the TST-positive group with a statistically significant mean difference of − 1.35% (95% CI: − 2.61 to − 0.10, *p* = 0.035). A similar finding was observed for %EWL, which was also lower in the TST-positive group at 1 year with a mean difference of − 2.87% (95% CI: − 5.63 to − 0.12, *p* = 0.041).

### Resolution of Obesity-related Diseases. (Table 5)

**Table 5:** One-year postoperative status of baseline obesity-related diseases in TST-positive and TST-negative participants with complete follow-up. Data reflect the proportion of patients achieving full resolution, partial improvement, or no change in status of obesity-related diseases at one year after metabolic and bariatric surgery. Between-group comparisons were conducted using fisher’s exact test. Statistically significant differences (*p* < 0.05) are shown in bold italic

Obesity-related disease	TST group	Resolution	Improved	Unchanged
Diabetes (*n* = 142)	Positive (*n* = 24)	24 (100.0)	0 (0.0)	0 (0.0)
	Negative (*n* = 118)	109 (92.4)	9 (7.6)	0 (0.0)
	*p* = 0.362			
Osteoarthritis (*n* = 244)	Positive (*n* = 43)	33 (76.7)	10 (23.3)	0 (0.0)
	Negative (*n* = 201)	0 (0.0)	201 (100.0)	0 (0.0)
	*p* < 0.001			
Dyslipidemia (*n* = 122)	Positive (*n* = 21)	21 (100.0)	0 (0.0)	0 (0.0)
	Negative (*n* = 101)	101 (100.0)	0 (0.0)	0 (0.0)
Hypertension (*n* = 249)	Positive (*n* = 44)	44 (100.0)	0 (0.0)	0 (0.0)
	Negative (*n* = 205)	205 (100.0)	0 (0.0)	0 (0.0)

Data are presented as numbers (percentages) within each category of obesity-related diseases. TST: Toxicology Screening Test; Table includes only participants with complete outcome data of obesity-related diseases at 1-year post-surgery. Resolution was defined as complete remission without the need for medical therapy; improvement indicated partial symptom relief or a reduction in medication. P-values are based on Fisher’s exact test for comparisons between the TST-positive and the TST-negative groups

Data demonstrated high remission rates for diabetes across both groups. In TST-positive patients, complete resolution was achieved at one year (100%), compared with 92.4% of TST-negative patients (*p* = 0.362).

For osteoarthritis, TST-positive patients experienced complete resolution in 76.7% and improvement in 23.3% of cases. In contrast, none of the 201 TST-negative patients achieved resolution. Outcomes for dyslipidemia and hypertension were 100% complete resolution across both groups.

### Supplementary Analyses

Multiple analyses were performed to assess the robustness of the primary findings and address potential confounding factors. Additional multivariable analyses evaluated the effects of sex and smoking status on postoperative outcomes using global longitudinal GEE models for pain scores and BMI, as well as linear regression models for anesthesia and opioid use, implemented in both unweighted and IPSW frameworks (ESM 1; Tables S2–S4). Furthermore, substance-specific analyses within the TST-positive group examined tramadol, cannabis, sedatives, and poly-substance exposure through descriptive comparisons and longitudinal GEE analyses (ESM 1; Tables S5, S6, and S8), as well as multivariable regression for anesthesia and opioid use (ESM 1; Table S7). These subgroup analyses are exploratory, given smaller strata, but they provide descriptive patterns and broadly consistent directionality with the main TST-positive versus TST-negative comparisons.

## Discussion

This prospective study highlights the prevalence and perioperative impact of SUD among MBS candidates in the MENA region. Preoperative TST identified recent substance users who would have gone undetected by self-reporting alone (12.1% of TST-positive cases were unreported), underscoring the limitations of history-based assessment, as noted by the discrepancy between self-reporting and screening test results. The use of rapid urine toxicological screening in this study offered a clinically pragmatic means of rapidly identifying recent substance exposure. Notably, the inclusion of tramadol and cannabis reflected the regional epidemiologic landscape of the MENA population [15, 27], where under-reported use remains of major clinical significance. The American Society of Regional Anesthesia and Pain Medicine (ASRA) [28] advocate for preoperative screening and inquiring about patients’ cannabinoid usage, dosage and frequency, method of administration, and the timing of their last use.

SUD disrupts the brain’s intrinsic circuitry related to reward and motivation, resulting in progressive neurological and physiological alterations that adversely affect decision-making and behavioral regulation. The observed association between TST positivity and higher anesthetic doses, postoperative pain, and opioid requirements highlights that even short-term exposure can significantly alter the perioperative anesthetic course despite the implementation of a multimodal analgesia. The exclusion of patients using sympathomimetic or alcohol-based substances ensured safety but likely attenuated the true magnitude of substance-related perioperative risk [15, 29–32].

### Prevalence and Clinical Relevance of SUD in MBS

Tramadol, cannabis, and benzodiazepines were the most frequently detected substances, reflecting regional SUD patterns distinct from those in Western cohorts. In contrast to Chao et al., who found benzodiazepines, opiates, and cotinine most common among U.S. MBS patients [17], tramadol and cannabis accounted for 57.9% and 36.8% of positive tests in our cohort; differences likely driven by prescribing practices and cultural factors in the MENA region.

The prevalence of positive TST in our cohort was 15.1%. Chao et al. documented a 12.7% positivity rate among 1,057 patients in their MBS cohort [17], while Menendez et al. reported a 19% positivity in patients before total joint arthroplasty, with 42% having unexpected positive results [33]. While Clavijo et al. [34] reported higher discrepancies between self-report and TST results in 88% of patients undergoing spine surgery. This discrepancy may be attributed to a small sample size and differences in study design, or the detected substances. The predominance of tramadol (52.6%) and cannabis (28.4%) aligns with rising regional use outlined by the United Nations Office on Drugs and Crime due to its accessibility and perceived safety [14, 35].

### Anesthesia, Recovery, and Postoperative Pain

The significantly higher doses of anesthetic drugs required by TST-positive patients, even after rigorous propensity-weighted adjustment for confounding factors, provide compelling evidence of pharmacodynamic tolerance and cross-tolerance. This finding is consistent with known neuroadaptive changes induced by chronic substance use, especially tramadol, including receptor down-regulation and desensitization, decreased pain threshold, and altered signal transduction pathways necessitating higher doses to achieve the desired response [36, 37]. Furthermore, chronic use of GABAergic substances, such as benzodiazepines, can induce cross-tolerance to propofol and volatile anesthetics through adaptations in GABA receptor subunits [38, 39]. ASRA guidelines acknowledge that chronic cannabis causes dose-dependent hyperalgesia and increased anesthetic requirements [28].

The TST-positive patients were characterized by significantly higher VAS scores and more morphine requirements, and the time to first postoperative opioid request was significantly shorter, supporting the presence of underlying tolerance and/or hyperalgesia. The prolonged opioid exposure causes Opioid-Induced Hyperalgesia (OIH) via central glutamatergic sensitization and descending facilitatory mechanisms [40, 41]. Our findings align with a growing body of literature and ASRA guidelines that chronic use of cannabinoids and tramadol may worsen postoperative pain and increase opioid use [28, 42]. The TST-positive cohort exhibited significantly prolonged recovery and higher agitation scores, as well as prolonged hospitalization. Postoperative emergence remains one of the least studied phenomena. This delay is likely multifactorial, from pharmacologic interactions between misused substances, anesthetics, and underlying neuropsychological alterations, with the abrupt cessation of chronic substance use in the perioperative period [12, 43–45].

The substance-stratified analysis suggested a trend toward higher anesthetic demands and prolonged recovery times and worse agitation scores in the poly-substance group compared to other groups, underscoring the multiple drug classes’ additive or synergistic neuroadaptations and the more pronounced interaction with anesthetics.

### Perioperative Morbidity and Underlying Mechanisms

TST-positive patients exhibited higher 30-day complication and readmission rates, underscoring a significant perioperative risk. Unlike Chao et al., who found no correlation between toxicology positivity and short-term outcomes [17]. Our findings align with Menendez et al., who reported increased complications in untreated substance users after arthroplasty. Notably, their study also suggested that preoperative treatment and intervention may mitigate the risks linked to SUD [33].

The mechanisms linking SUD to complications are multifactorial and biologically plausible. Chronic psychoactive substance use impairs immune function, angiogenesis, and hepatic enzyme activity, compromising wound healing [46]. Increased pain and opioid needs may also delay mobilization, contributing to pulmonary or thromboembolic events. A large U.S. cohort of over one million abdominal surgeries linked SUD with higher overall complications and markedly elevated infectious and respiratory risks [47]. Such concordance reinforces that SUD is an independent determinant of perioperative vulnerability and higher morbidity across surgical populations.

Beyond SUD, smoking likely compounds this vulnerability. Nicotine increases airway reactivity, impairs oxygenation, and delays wound healing [22, 48, 49]. Although smoking status was recorded and incorporated into perioperative planning, the potential synergistic effect of tobacco exposure may have contributed to the higher early morbidity seen in TST-positive patients.

### Weight Loss Outcomes

Both groups experienced marked reductions in body weight and BMI over time, with no statistically significant differences. Although %TWL and %EWL were statistically lower in the TST-positive group at 12 months, the between-group differences were small in magnitude and should not be overstated. Given the large sample size, statistical significance may reflect a small effect size, and residual confounding cannot be excluded. Nonetheless, TST-positivity may identify patients who could benefit from enhanced postoperative monitoring and support.

Several factors may explain this attenuation, such as substance-related physiological effects, such as cannabis-associated hyperphagia or sedative-induced energy reduction [50, 51], higher burden of psychiatric comorbidity, and lack of follow-up [52, 53]. Recent MBS cohorts reported comparable %EWL, %TWL, and complication rates between cannabis users and non-users [17].

### Clinical Implications and Strengths

The prospective observational design enabled a standardized and detailed assessment of anesthetic, surgical, and metabolic outcomes, allowing a clear understanding of the influence of substance use on perioperative and short-term postoperative outcomes following MBS. The large sample size provided sufficient statistical power to detect clinically meaningful differences and to perform robust subgroup analyses.

IPSW was applied to balance baseline characteristics, minimize confounding, and strengthen the internal validity of the observed associations. The broad outcome framework, encompassing anesthetic requirements, recovery parameters, pain intensity, opioid consumption, postoperative complications, readmissions, and one-year weight loss and resolution of obesity-related diseases, provided an integrated evaluation of both perioperative and long-term follow-up effects, thereby minimizing attrition bias.

The choice of rapid urine immunoassay represents a major strength, balancing detection window, specificity, and cost-effectiveness. While some studies have used serum analysis [17] or confirmatory techniques such as liquid chromatography–tandem mass spectrometry (LC-MS/MS) [54]. These approaches, although highly specific, are costly and time-consuming, rendering them impractical for routine screening in high-volume surgical settings.

The seven-substance panel (especially tramadol and cannabis) was purposefully selected to reflect both pharmacological and epidemiological relevance in the MENA region [15, 27] and by their known capacity to alter anesthetic and analgesic requirements.

Patients dependent on acute sympathomimetics, cocaine, amphetamines, and alcohol were strategically excluded based on perioperative risks and anesthetic interaction: severe hemodynamic instability, arrhythmias, hypertension, and myocardial ischemia [15, 29, 30], whereas alcohol dependence predisposes to withdrawal syndromes, nutritional deficiencies, hepatic dysfunction, and poor surgical compliance [31, 32].

Collectively, the authors support and advocate for the integration of routine preoperative toxicological screening into MBS pathways, particularly in regions where self-reported substance use may be unreliable. The anesthetist should plan for a tailored perioperative and empathetic, individualized approach protocol for the TST-positive individuals, featuring anesthetic pre-planning, multimodal analgesia providing effective pain relief [28, 55], prioritizing non-opioid adjuvants to mitigate OIH and reduce the risk of respiratory depression while minimizing drug-seeking behavior and cultural fears related to pain or withdrawal, ensuring a safer and enhanced recovery.

### Limitations

This study has notable limitations that affect interpretation. Its observational design restricts causal inference about substance exposure and perioperative outcomes. Although IPSW aims to address baseline imbalances, residual confounding from unmeasured factors like socioeconomic status and health literacy may still influence SUD patterns, anesthetic, and surgical outcomes in the MENA region. Exclusions of patients with sympathomimetic use or chronic alcohol dependence likely led to an underestimation of the impact. Additionally, while smoking status was analyzed, residual confounding remains due to its effects on anesthetic pharmacokinetics and outcomes. The toxicological assay imposes limitations; although clinically practical, it cannot quantify dose or chronicity or rule out false positives/negatives without confirmatory testing (e.g., LC-MS/MS). Furthermore, short detection windows for rapidly metabolized substances like cocaine may result in under-detection, and the qualitative nature of the urine dipstick assay hampers the quantification of exposure. Finally, the one-year follow-up curtails long-term assessments, and the findings from this single-center study in the MENA region may not be generalized to other populations, highlighting the diverse substance-use profiles and healthcare pathways.

#### Future Directions

Future research should focus on 3–5-year longitudinal studies to evaluate the long-term effects of preoperative substance misuse, such as alcohol and cocaine, on outcomes after MBS. Key areas include the effectiveness of cessation programs, personalized perioperative management for those with positive toxicology results, and exploring sociocultural barriers to substance use disclosure in the MENA population to develop culturally sensitive interventions.

## Conclusion

This large prospective study demonstrates that preoperative toxicological screening identifies a substantial proportion of MBS candidates with undisclosed SUD, which is associated with a more challenging perioperative anesthetic course, increased anesthetic requirements, delayed recovery, higher postoperative pain, and greater risk for complications. Despite these anesthetic challenges, the metabolic and weight loss benefits at one year remained preserved. Routine preoperative toxicology screening enhances perioperative anesthetic planning to optimize safety and outcomes, particularly in regions where substance use is underreported. Figure 1: Participant flowchart showing recruitment and follow-up..

## Supplementary Information

Below is the link to the electronic supplementary material.

Supplementary Material 1

## Data Availability

The datasets generated and analyzed during the current study are available from the corresponding author upon reasonable request.
