# Supplementary material for: Impact of Preoperative Toxicological Screening on Perioperative Anesthetic Management and Short-Term Outcomes Following Metabolic and Bariatric Surgery: A Prospective Observational Study
Source: Obes Surg. 2026 Mar 31;36(5):2132–49. doi: 10.1007/s11695-026-08534-3 (PMC13222261; doi:10.1007/s11695-026-08534-3)
Supplement: Supplementary file 1 — Supplementary Material 1 [file 11695_2026_8534_MOESM1_ESM.docx]

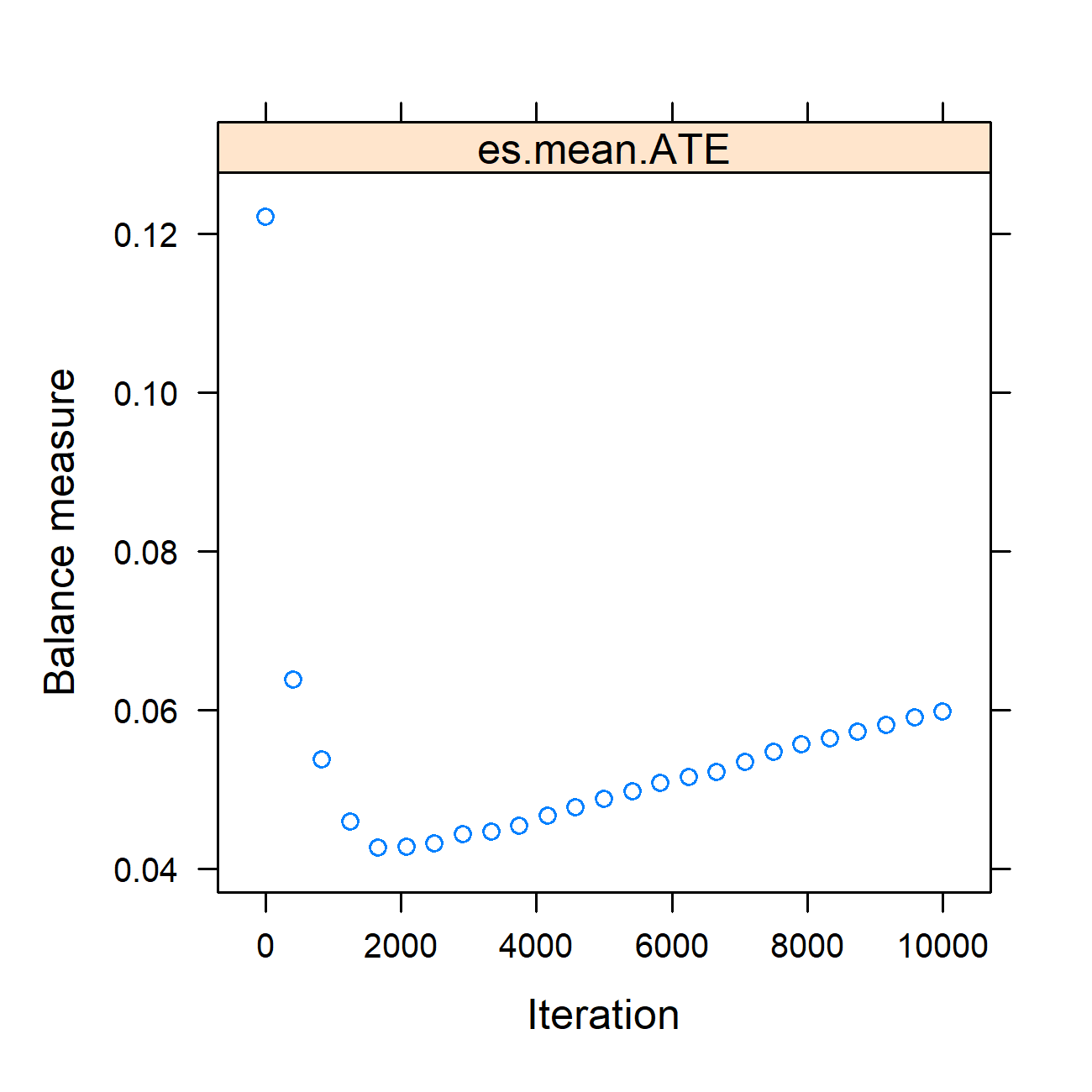


**Figure S1:** Trend of balance measures across iterations in the GBM model for Inverse Probability of Treatment Weighting (IPSW). This figure represent the optimization plot of the average absolute standardized effect size (es.mean.ATE) across boosting iterations during propensity score estimation using the twang package. The Y-axis represents the balance measure (lower is better), and the X-axis shows the number of boosting iterations. Using the `es.mean` stopping rule, the optimal covariate balance was achieved at 1750 iterations, where the average standardized effect size reached its minimum under the Average Treatment Effect (ATE) estimand.


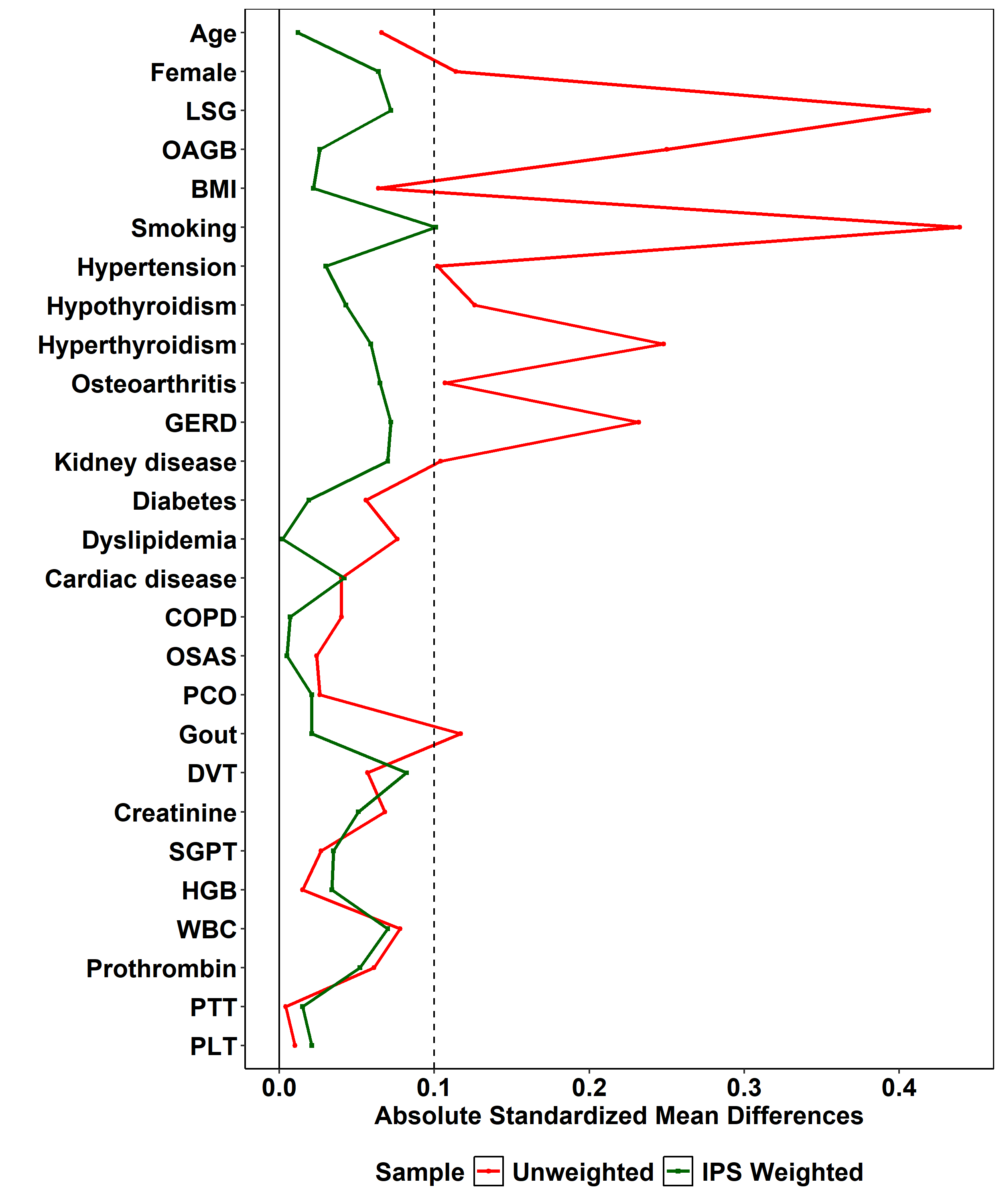


**Figure S2:** Love Plot illustrates the effectiveness of IPS weighting in balancing covariates between treatment groups. The green line represents the SMDs in the IPS weighted sample, showing significant reductions in discrepancies across most variables compared to the red line, which represents the unweighted sample. The vertical dotted line at 0.1 denotes the threshold below which the SMDs are generally considered negligible, indicating good balance.


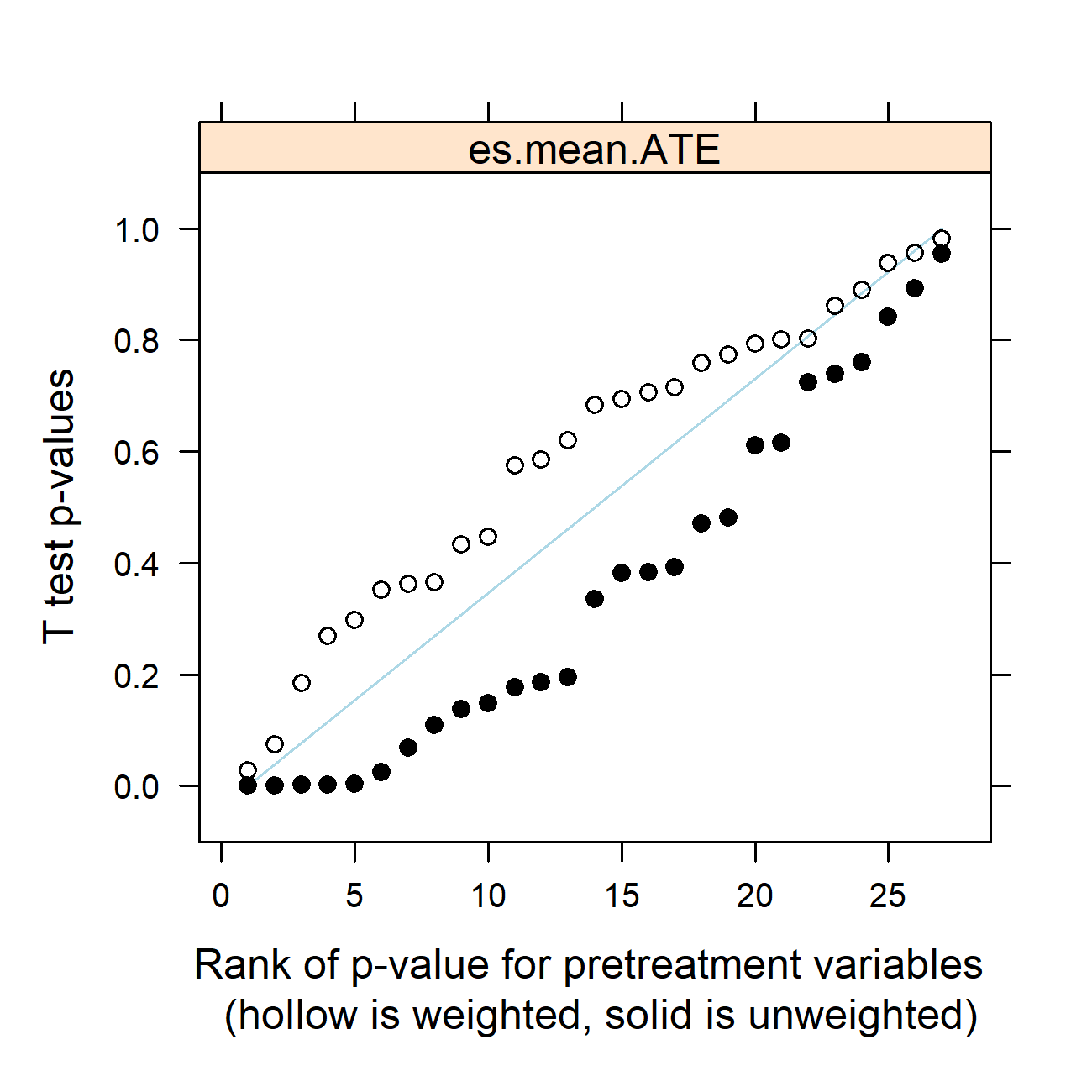


**Figure S3.** Rank of t-test p-values for pretreatment variables in the study, comparing IPS weighted (hollow circles) and unweighted (solid circles) samples. This plot visualizes the improvement in p-values due to IPS weighting, indicating a more uniform distribution across the weighted sample.


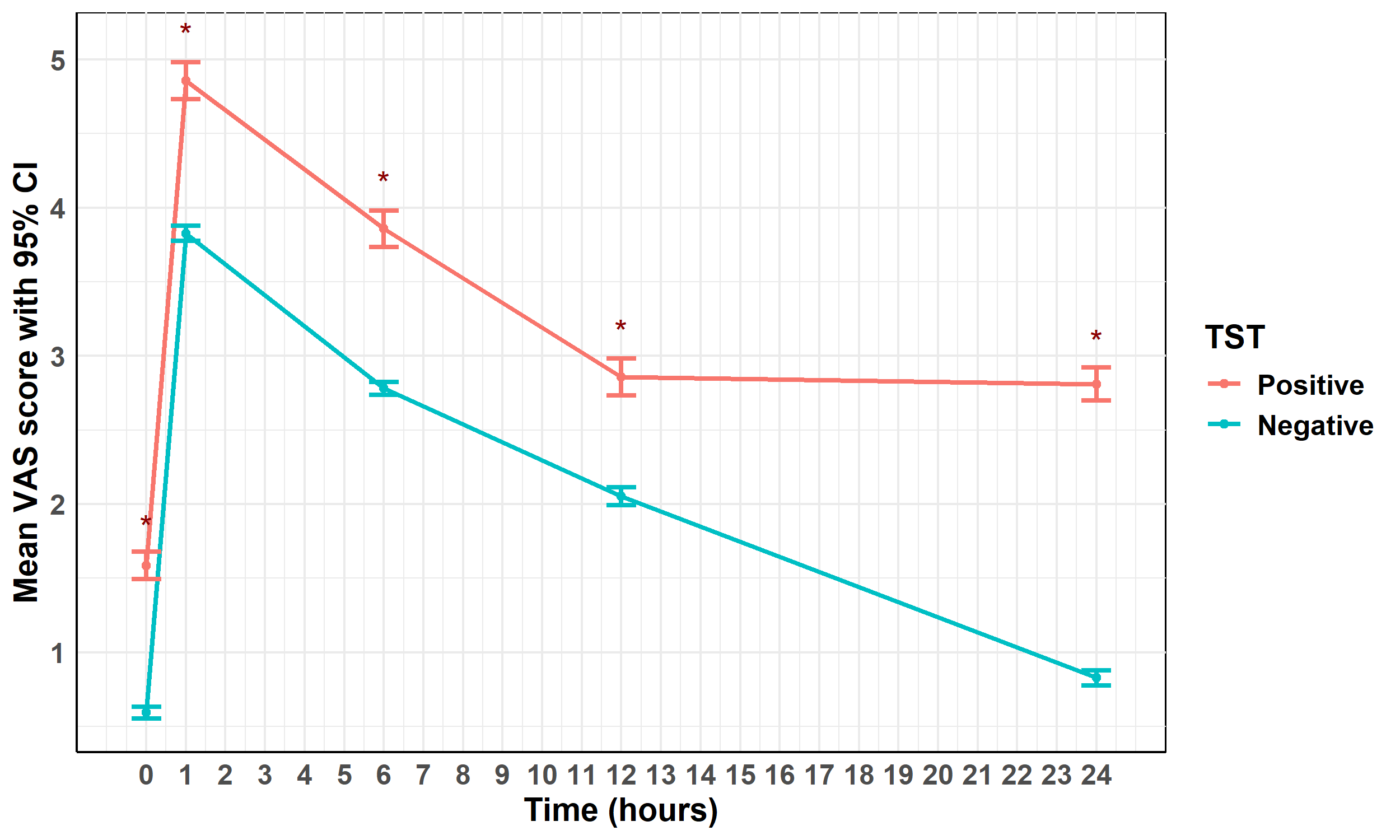


**Figure S4:** Unadjusted VAS Pain Scores Over 24 Hours by TST Group. The graph illustrates the unadjusted mean Visual Analog Scale (VAS) pain scores with 95% confidence intervals at baseline and at 1, 6, 12, and 24 hours postoperatively for TST-positive and TST-negative participants. Pain scores peaked at 1 hour in both groups and gradually declined over time. At each time point, mean VAS scores were consistently higher in the TST-positive group. Asterisks (*) denote statistically significant differences between groups at the corresponding time point (p < 0.05). These findings represent unadjusted comparisons; adjusted results are provided in Figure 2.


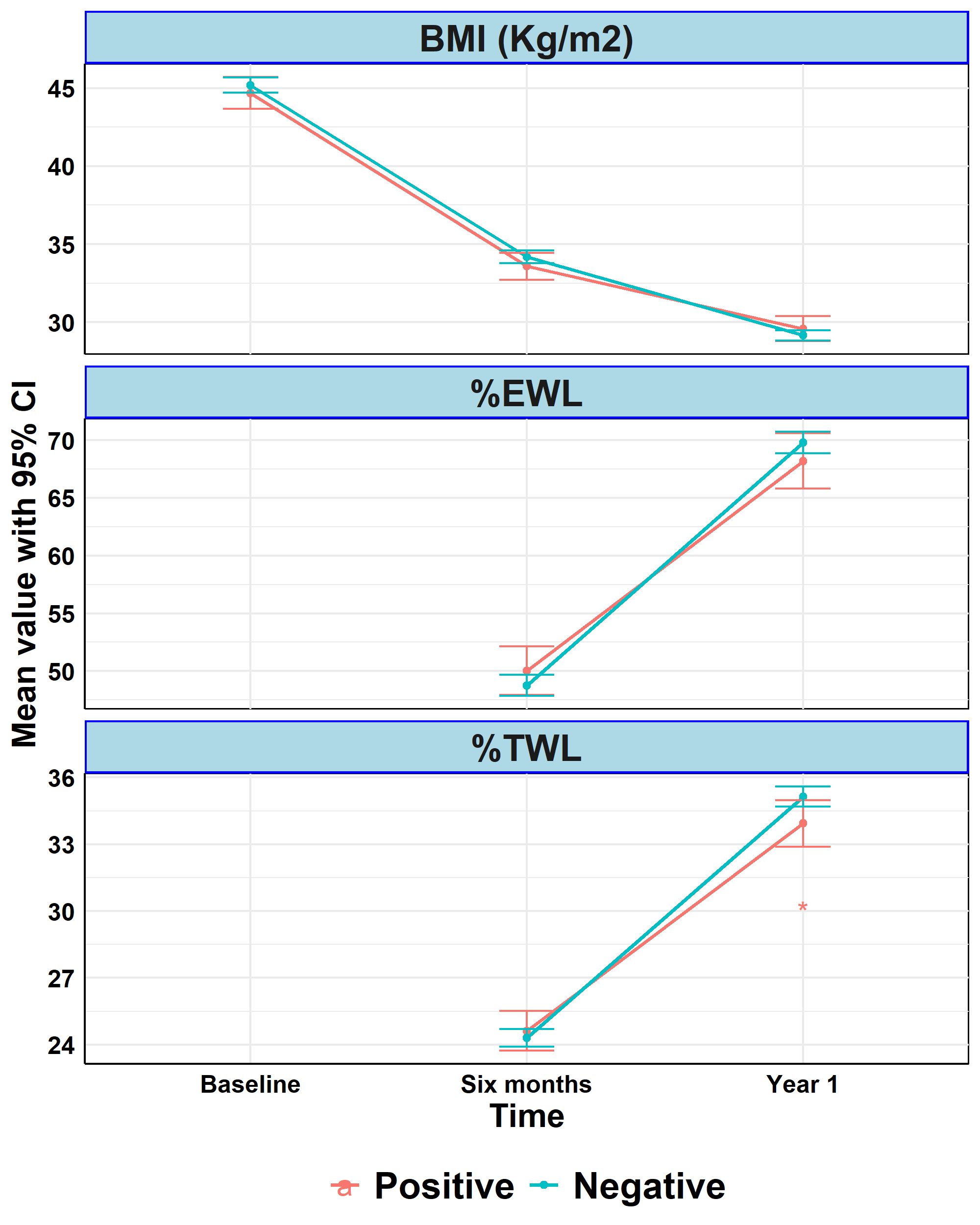


**Figure S5:** Unadjusted Changes in BMI, %EWL, and %TWL Over One Year by TST Group. The graphs presents the unadjusted mean values and 95% confidence intervals for body mass index (BMI), percent excess weight loss (%EWL), and percent total weight loss (%TWL) at baseline, 6 months, and 1 year after surgery, stratified by TST group. Both groups showed marked reductions in BMI and progressive increases in %EWL and %TWL over time. At the 1-year follow-up, the TST-negative group exhibited slightly higher %TWL compared to the TST-positive group. Asterisks (*) denote statistically significant between-group differences at the corresponding time point (p < 0.05). These results represent the unadjusted analysis; adjusted comparisons are shown in Figure 5.

| **Table S1:** Absolute standardized mean differences (SMD) for baseline covariates before and after inverse propensity score weighting (IPSW) between TST-positive and TST-negative participants | | | | |
| --- | --- | --- | --- | --- |
| **Variable** | **Before Weighting** | | **After Weighting** | |
|  | **aSMD** | **p** | **aSMD** | **p** |
| Age | 0.07 | 0.392 | 0.01 | 0.889 |
| Female | 0.11 | 0.149 | 0.06 | 0.447 |
| SG | 0.42 | ***< 0.001*** | 0.07 | 0.297 |
| OAGB | 0.25 | ***0.002*** | 0.03 | 0.705 |
| BMI pre | 0.06 | 0.382 | 0.02 | 0.773 |
| Smoking | 0.44 | ***< 0.001*** | 0.10 | 0.184 |
| Hypertension | 0.10 | 0.195 | 0.03 | 0.715 |
| Hypothyroidism | 0.13 | 0.109 | 0.04 | 0.585 |
| Hyperthyroidism | 0.25 | 0.002 | 0.06 | 0.366 |
| Osteoarthritis | 0.11 | 0.177 | 0.07 | 0.432 |
| GERD | 0.23 | 0.003 | 0.07 | 0.351 |
| Kidney | 0.10 | 0.186 | 0.07 | 0.363 |
| Diabetes | 0.06 | 0.481 | 0.02 | 0.803 |
| Dyslipidemia | 0.08 | 0.336 | 0.00 | 0.981 |
| Cardiac | 0.04 | 0.611 | 0.04 | 0.619 |
| COPD | 0.04 | 0.615 | 0.01 | 0.937 |
| OSAS | 0.02 | 0.760 | 0.01 | 0.956 |
| DVT | 0.06 | 0.471 | 0.08 | 0.269 |
| Creatine pre | 0.07 | 0.384 | 0.05 | 0.574 |
| SGPT pre | 0.03 | 0.723 | 0.04 | 0.693 |
| HGB pre | 0.02 | 0.842 | 0.03 | 0.683 |
| WBC pre | 0.08 | 0.024 | 0.07 | 0.028 |
| Prothrombin pre | 0.06 | 0.068 | 0.05 | 0.074 |
| PTT pre | 0.00 | 0.955 | 0.02 | 0.862 |
| PLT pre | 0.01 | 0.893 | 0.02 | 0.793 |

Absolute standardized mean differences (aSMD) and corresponding p values are shown for key baseline variables before and after inverse propensity score weighting (IPSW) under the average treatment effect (ATE) framework. Lower aSMD values indicate improved covariate balance between TST-positive and TST-negative groups, with values < 0.10 generally considered indicative of adequate balance. Post-weighting results demonstrate substantial improvement in balance across measured covariates, including sex and smoking status. GEE, generalized estimating equation; VAS, visual analogue scale; MD, mean difference; CI, confidence interval; IPSW, inverse propensity score weighting; TST, toxicology screening test

| **Supplementary Table S2:** Global longitudinal generalized estimating equation (GEE) analysis of postoperative VAS pain scores, including the overall effect of TST positivity and adjusting for sex and smoking status | | | | |
| --- | --- | --- | --- | --- |
| **Factor** | **Unweighted** | | **IPSW** | |
|  | **MD (95% CI)** | **p** | **MD (95% CI)** | **p** |
| **Time** |  |  |  |  |
| Baseline | Reference |  | Reference |  |
| 1 hour | 3.24 (3.22, 3.26) | ***< 0.001*** | 3.25 (3.22, 3.28) | ***< 0.001*** |
| 6 hours | 2.20 (2.18, 2.22) | ***< 0.001*** | 2.22 (2.19, 2.26) | ***< 0.001*** |
| 12 hours | 1.43 (1.40, 1.47) | ***< 0.001*** | 1.37 (1.33, 1.41) | ***< 0.001*** |
| 24 hours | 0.38 (0.35, 0.41) | ***< 0.001*** | 0.66 (0.61, 0.71) | ***< 0.001*** |
| **TST** |  |  |  |  |
| Negative | Reference |  | Reference |  |
| Positive | 1.18 (1.06, 1.30) | ***< 0.001*** | 1.20 (1.07, 1.32) | ***< 0.001*** |
| **Smoking** |  |  |  |  |
| No | Reference |  | Reference |  |
| Yes | -0.02 (-0.14, 0.11) | 0.796 | -0.07 (-0.21, 0.08) | 0.360 |
| **Sex** |  |  |  |  |
| Male | Reference |  | Reference |  |
| Female | -0.05 (-0.15, 0.05) | 0.298 | -0.31 (-0.45, -0.17) | ***< 0.001*** |

Values are reported as mean differences (MD) with 95% confidence intervals (CI) and corresponding p values. Postoperative Visual Analog Scale (VAS) pain scores measured at baseline, 1 hour, 6 hours, 12 hours, and 24 hours were analyzed using generalized estimating equations (GEE) with an exchangeable working correlation structure to account for within-subject correlation. Time was modeled as a categorical variable with baseline as the reference category. The TST effect represents the overall marginal difference in VAS scores averaged across all time points, adjusted for sex and smoking status, which were included to address potential confounding. Unweighted and inverse propensity score–weighted (IPSW) analyses under the average treatment effect (ATE) framework are shown. Statistically significant results (p < 0.05) are indicated in bold italic. MD, mean difference; CI, confidence interval; IPSW, inverse propensity score weighting; TST, toxicology screening test.

| **Table S3:** Unweighted and inverse propensity score–weighted linear regression analyses of anesthesia requirements and early postoperative outcomes after Metabolic and bariatric surgery (MBS), assessing the overall effect of TST positivity and adjusting for sex and smoking. | | | | |
| --- | --- | --- | --- | --- |
| **Factor** | **Unweighted** | | **IPSW** | |
|  | **MD (95% CI)** | **p** | **MD (95% CI)** | **p** |
| **Propofol Dose (mg)** | | | | |
| **TST** |  |  |  |  |
| Negative | Reference |  | Reference |  |
| Positive | 48.22 (45.98, 50.46) | ***< 0.001*** | 49.53 (46.19, 52.87) | ***< 0.001*** |
| **Smoking** |  |  |  |  |
| No | Reference |  | Reference |  |
| Yes | -0.46 (-2.59, 1.66) | 0.669 | -2.65 (-6.22, 0.93) | 0.147 |
| **Sex** |  |  |  |  |
| Male | Reference |  | Reference |  |
| Female | -1.91 (-3.74, -0.08) | ***0.040*** | -8.38 (-11.88, -4.88) | ***< 0.001*** |
| **Intraoperative Fentanyl Dose (mcg)** | | | | |
| **TST** |  |  |  |  |
| Negative | Reference |  | Reference |  |
| Positive | 49.92 (47.44, 52.40) | ***< 0.001*** | 51.10 (48.67, 53.53) | ***< 0.001*** |
| **Smoking** |  |  |  |  |
| No | Reference |  | Reference |  |
| Yes | -1.01 (-3.36, 1.34) | 0.399 | -2.39 (-5.14, 0.36) | 0.088 |
| **Sex** |  |  |  |  |
| Male | Reference |  | Reference |  |
| Female | -1.12 (-3.14, 0.90) | 0.278 | -5.95 (-8.62, -3.28) | ***< 0.001*** |
| **Perioperative Morphine Dose (mg)** | | | | |
| **TST** |  |  |  |  |
| Negative | Reference |  | Reference |  |
| Positive | 3.46 (3.17, 3.74) | ***< 0.001*** | 3.54 (3.26, 3.82) | ***< 0.001*** |
| **Smoking** |  |  |  |  |
| No | Reference |  | Reference |  |
| Yes | -0.08 (-0.35, 0.19) | 0.577 | -0.21 (-0.55, 0.12) | 0.211 |
| **Sex** |  |  |  |  |
| Male | Reference |  | Reference |  |
| Female | -0.21 (-0.45, 0.02) | 0.073 | -0.80 (-1.12, -0.48) | ***< 0.001*** |
| **Recovery Time (min)** | | | | |
| **TST** |  |  |  |  |
| Negative | Reference |  | Reference |  |
| Positive | 5.13 (4.62, 5.64) | ***< 0.001*** | 5.34 (4.84, 5.84) | ***< 0.001*** |
| **Smoking** |  |  |  |  |
| No | Reference |  | Reference |  |
| Yes | -0.14 (-0.63, 0.34) | 0.561 | -0.45 (-1.02, 0.12) | 0.12 |
| **Sex** |  |  |  |  |
| Male | Reference |  | Reference |  |
| Female | -0.22 (-0.63, 0.20) | 0.303 | -1.13 (-1.67, -0.60) | ***< 0.001*** |
| **Recovery Status score** | | | | |
| **TST** |  |  |  |  |
| Negative | Reference |  | Reference |  |
| Positive | 2.44 (2.28, 2.60) | ***< 0.001*** | 2.48 (2.30, 2.65) | ***< 0.001*** |
| **Smoking** |  |  |  |  |
| No | Reference |  | Reference |  |
| Yes | -0.06 (-0.22, 0.09) | 0.422 | -0.09 (-0.28, 0.09) | 0.329 |
| **Sex** |  |  |  |  |
| Male | Reference |  | Reference |  |
| Female | -0.07 (-0.20, 0.06) | 0.286 | -0.31 (-0.50, -0.13) | ***0.001*** |
| **First Opioid Request** | | | | |
| **TST** |  |  |  |  |
| Negative | Reference |  | Reference |  |
| Positive | -1.05 (-1.26, -0.84) | ***< 0.001*** | -0.95 (-1.15, -0.76) | ***< 0.001*** |
| **Smoking** |  |  |  |  |
| No | Reference |  | Reference |  |
| Yes | -0.16 (-0.36, 0.04) | 0.109 | -0.27 (-0.50, -0.04) | ***0.019*** |
| **Sex** |  |  |  |  |
| Male | Reference |  | Reference |  |
| Female | 0.08 (-0.09, 0.25) | 0.363 | 0.00 (-0.20, 0.20) | 0.977 |

Results are presented as mean differences (MD) with 95% confidence intervals (CI) and corresponding p values. Separate linear regression models were fitted for each outcome to estimate the overall effect of TST positivity on anesthesia requirements, opioid consumption, recovery characteristics, and timing of first postoperative opioid request. Models included sex and smoking status as covariates to address potential confounding. Both unweighted analyses and inverse propensity score–weighted (IPSW) analyses under the average treatment effect (ATE) framework are shown. IPSW estimates reflect the weighted pseudo-population. Statistically significant results (p < 0.05) are indicated in bold italic. GEE, generalized estimating equation; BMI, body mass index; MD, mean difference; CI, confidence interval; IPSW, inverse propensity score weighting; TST, toxicology screening test.

| **Table S4:** Global longitudinal generalized estimating equation (GEE) analysis of changes in body mass index (BMI) after Metabolic and bariatric surgery (MBS), including the overall effect of TST positivity and adjusting for sex and smoking status | | | | |
| --- | --- | --- | --- | --- |
| **Factor** | **Unweighted** | | **IPSW** | |
|  | **MD (95% CI)** | **p** | **MD (95% CI)** | **p** |
| **Time** |  |  |  |  |
| Baseline | Reference |  | Reference |  |
| 6 months | -10.99 (-11.19, -10.80) | ***< 0.001*** | -11.09 (-11.35, -10.84) | ***< 0.001*** |
| 1 year | -15.92 (-16.20, -15.64) | ***< 0.001*** | -15.74 (-16.09, -15.39) | ***< 0.001*** |
| **TST** |  |  |  |  |
| Negative | Reference |  | Reference |  |
| Positive | 0.16 (-0.77, 1.09) | 0.734 | 0.38 (-0.58, 1.34) | 0.437 |
| **Smoking** |  |  |  |  |
| No | Reference |  | Reference |  |
| Yes | -2.65 (-3.52, -1.79) | ***< 0.001*** | -2.87 (-3.88, -1.85) | ***< 0.001*** |
| **Sex** |  |  |  |  |
| Male | Reference |  | Reference |  |
| Female | -0.82 (-1.69, 0.06) | 0.068 | -1.14 (-2.19, -0.08) | ***0.034*** |

Results are presented as mean differences (MD) with 95% confidence intervals (CI) and corresponding p values. Body mass index (BMI) measured preoperatively, at 6 months, and at 1 year postoperatively was analyzed using generalized estimating equations (GEE) with an exchangeable working correlation structure to account for within-subject correlation. Time was modeled as a categorical variable with preoperative BMI as the reference category. The TST effect represents the overall marginal difference in BMI averaged across follow-up time points, adjusted for sex and smoking status, which were included to address potential confounding. Both unweighted and inverse propensity score–weighted (IPSW) analyses under the average treatment effect (ATE) framework are shown. Statistically significant results (p < 0.05) are indicated in bold italic. SE, standard error; TST, toxicology screening test; BMI, body mass index; VAS, visual analogue scale; TWL, total weight loss; EWL, excess weight loss; OSAS, obstructive sleep apnea syndrome; GERD, gastroesophageal reflux disease; DVT, deep venous thrombosis; UTI, urinary tract infection; FBS, fasting blood sugar; HbA1c, glycated hemoglobin; TSH, thyroid-stimulating hormone; T3, triiodothyronine; T4, thyroxine; INR, international normalized ratio; HGB, hemoglobin; WBC, white blood cell count; PLT, platelet count; PTT, partial thromboplastin time; SGOT, aspartate aminotransferase (AST); SGPT, alanine aminotransferase (ALT); LSG, laparoscopic sleeve gastrectomy; OAGB, one-anastomosis gastric bypass; RYGB, Roux-en-Y gastric bypass; PCO, polycystic ovary syndrome.

| **Table S5:** Substance-specific comparison of baseline characteristics, perioperative variables, postoperative pain, and weight-loss outcomes among TST-positive patients. | | | | | |
| --- | --- | --- | --- | --- | --- |
| **Variable** | **Tramadol only**  **(n = 102)** | **Cannabis only**  **(n = 65)** | **Sedatives ± Opiates**  **(n = 15)** | **Poly-substance**  **(n = 8)** | **p** |
| **Age** | 36.5 ± 1.1 | 39.3 ± 1.4 | 35.5 ± 3.1 | 39.4 ± 2.2 | 0.277 |
| **Sex** |  |  |  |  |  |
| Female | 34 (33.3) | 24 (36.9) | 3 (20.0) | 3 (37.5) | 0.670 |
| Male | 68 (66.7) | 41 (63.1) | 12 (80.0) | 5 (62.5) |  |
| **Anthropometrics** |  |  |  |  |  |
| Height | 166.1 ± 1.0 | 166.3 ± 1.5 | 164.9 ± 2.2 | 166.9 ± 2.9 | 0.891 |
| Weight | 122.7 ± 2.4 | 122.7 ± 3.3 | 127.8 ± 6.6 | 140.6 ± 7.9 | 0.188 |
| BMI | 44.3 ± 0.7 | 44.0 ± 0.8 | 46.8 ± 1.9 | 50.8 ± 3.2 | 0.095 |
| **Type of MBS** |  |  |  |  |  |
| LSG | 91 (89.2) | 49 (75.4) | 12 (80.0) | 7 (87.5) | 0.169 |
| OAGB | 8 (7.8) | 10 (15.4) | 1 (6.7) | 1 (12.5) |  |
| RYGB | 3 (2.9) | 6 (9.2) | 2 (13.3) | 0 (0.0) |  |
| **Operative time (min)** | 47.3 ± 0.5 | 47.7 ± 0.5 | 48.3 ± 1.1 | 48.2 ± 1.4 | 0.823 |
| **Smoking** | 27 (26.5) | 35 (53.8) | 2 (13.3) | 1 (12.5) | ***< 0.001*** |
| **Self-reported substance use** |  |  |  |  |  |
| No | 13 (12.7) | 7 (10.8) | 1 (6.7) | 2 (25.0) | 0.580 |
| Yes | 89 (87.3) | 58 (89.2) | 14 (93.3) | 6 (75.0) |  |
| **Associated Medical illnesses** |  |  |  |  |  |
| OSAS | 50 (49.0) | 32 (49.2) | 5 (33.3) | 5 (62.5) | 0.594 |
| Hypertension | 26 (25.5) | 15 (23.1) | 2 (13.3) | 4 (50.0) | 0.295 |
| Osteoarthritis | 21 (20.6) | 20 (30.8) | 4 (26.7) | 1 (12.5) | 0.450 |
| Diabetes | 16 (15.7) | 9 (13.8) | 0 (0.0) | 1 (12.5) | 0.435 |
| Hypothyroidism | 16 (15.7) | 7 (10.8) | 3 (20.0) | 1 (12.5) | 0.686 |
| Dyslipidemia | 13 (12.7) | 9 (13.8) | 0 (0.0) | 1 (12.5) | 0.523 |
| Insulin resistance | 5 (4.9) | 7 (10.8) | 1 (6.7) | 0 (0.0) | 0.429 |
| GERD | 12 (11.8) | 10 (15.4) | 0 (0.0) | 1 (12.5) | 0.426 |
| PCO | 7 (6.9) | 0 (0.0) | 1 (6.7) | 0 (0.0) | 0.112 |
| Cardiac | 4 (3.9) | 1 (1.5) | 1 (6.7) | 1 (12.5) | 0.214 |
| DVT | 2 (2.0) | 1 (1.5) | 0 (0.0) | 0 (0.0) | 1.000 |
| Gout | 4 (3.9) | 2 (3.1) | 0 (0.0) | 0 (0.0) | 1.000 |
| Hyperthyroidism | 2 (2.0) | 3 (4.6) | 0 (0.0) | 1 (12.5) | 0.282 |
| Kidney | 2 (2.0) | 1 (1.5) | 0 (0.0) | 0 (0.0) | 1.000 |
| **Lab Investigations** |  |  |  |  |  |
| HGB | 12.9 ± 0.2 | 13.1 ± 0.2 | 12.8 ± 0.2 | 13.2 ± 0.4 | 0.840 |
| PLT | 285.4 ± 6.7 | 307.4 ± 9.7 | 316.9 ± 20.5 | 294.9 ± 17.9 | 0.333 |
| WBC | 7.6 ± 0.3 | 7.6 ± 0.3 | 8.5 ± 0.5 | 7.5 ± 0.5 | 0.300 |
| Clotting time | 416.7 ± 11.2 | 448.6 ± 15.5 | 472.5 ± 51.0 | 395.0 ± 33.3 | 0.269 |
| Bleeding time | 130.0 ± 7.1 | 150.4 ± 12.0 | 136.7 ± 22.3 | 139.4 ± 20.0 | 0.824 |
| Prothrombin | 11.9 ± 0.1 | 12.1 ± 0.1 | 11.7 ± 0.2 | 11.7 ± 0.4 | 0.619 |
| Prothrombin Activity | 95.8 ± 0.9 | 94.8 ± 1.1 | 95.3 ± 2.4 | 98.2 ± 5.4 | 0.540 |
| PTT | 31.7 ± 0.4 | 30.5 ± 0.6 | 28.3 ± 1.0 | 31.1 ± 1.1 | 0.061 |
| INR | 1.0 ± 0.0 | 1.0 ± 0.0 | 1.0 ± 0.0 | 1.0 ± 0.0 | 0.203 |
| Urea | 27.4 ± 0.8 | 26.2 ± 0.9 | 26.9 ± 2.1 | 24.0 ± 1.6 | 0.631 |
| Creatinine | 0.8 ± 0.0 | 0.8 ± 0.0 | 0.7 ± 0.1 | 0.9 ± 0.1 | 0.373 |
| SGOT | 22.9 ± 1.3 | 21.6 ± 1.3 | 20.9 ± 1.6 | 21.3 ± 2.9 | 0.975 |
| SGPT | 24.2 ± 1.4 | 24.4 ± 1.3 | 24.7 ± 2.9 | 26.9 ± 6.1 | 0.728 |
| FBS | 97.1 ± 2.4 | 102.8 ± 4.8 | 94.6 ± 6.2 | 93.4 ± 4.8 | 0.806 |
| HBA1C | 5.7 ± 0.1 | 5.7 ± 0.1 | 5.5 ± 0.2 | 5.4 ± 0.3 | 0.913 |
| Triglycerides | 140.0 ± 5.8 | 135.0 ± 7.5 | 149.6 ± 13.0 | 119.3 ± 17.4 | 0.440 |
| Cholesterol | 173.9 ± 4.4 | 164.3 ± 6.0 | 198.8 ± 8.6 | 146.8 ± 15.5 | 0.010 |
| TSH | 2.0 ± 0.1 | 2.1 ± 0.2 | 2.5 ± 0.4 | 2.3 ± 0.3 | 0.332 |
| Free T3 | 3.2 ± 0.1 | 3.0 ± 0.1 | 3.1 ± 0.2 | 3.6 ± 0.2 | 0.206 |
| Free T4 | 1.2 ± 0.1 | 1.5 ± 0.2 | 1.1 ± 0.1 | 1.3 ± 0.1 | 0.221 |
| Hepatitis B positive | 1 (1.0) | 1 (1.5) | 0 (0.0) |  | 1.000 |
| Hepatitis C positive | 0 (0.0) | 0 (0.0) | 0 (0.0) |  |  |
| **Operative data** |  |  |  |  |  |
| hospital stay | 2.1 ± 0.0 | 2.1 ± 0.1 | 2.1 ± 0.1 | 2.0 ± 0.0 | 0.885 |
| propofol dose mg | 213.2 ± 2.4 | 214.3 ± 2.9 | 220.0 ± 6.5 | 232.5 ± 5.3 | 0.134 |
| intra fentanyl dose mcg | 173.5 ± 1.6 | 174.1 ± 2.0 | 178.0 ± 4.8 | 183.8 ± 4.2 | 0.264 |
| Peri morphine dose mg | 6.0 ± 0.2 | 6.2 ± 0.2 | 6.4 ± 0.6 | 7.0 ± 0.7 | 0.502 |
| Recovery time min | 14.9 ± 0.3 | 15.0 ± 0.4 | 16.4 ± 0.9 | 17.2 ± 0.8 | 0.110 |
| Recovery status score | 2.0 ± 0.1 | 2.0 ± 0.1 | 0.5 ± 0.4 | 2.6 ± 0.2 | ***< 0.001*** |
| First opioid request | 3.5 ± 0.1 | 1.2 ± 0.0 | 2.7 ± 0.3 | 1.4 ± 0.2 | ***< 0.001*** |
| **VAS** |  |  |  |  |  |
| Baseline | 1.5 ± 0.1 | 1.6 ± 0.1 | 1.5 ± 0.2 | 2.0 ± 0.2 | 0.213 |
| One hour | 4.8 ± 0.1 | 4.8 ± 0.1 | 4.9 ± 0.2 | 5.2 ± 0.3 | 0.541 |
| Six hours | 3.8 ± 0.1 | 3.8 ± 0.1 | 3.9 ± 0.2 | 4.2 ± 0.3 | 0.541 |
| Twelve hours | 2.8 ± 0.1 | 2.8 ± 0.1 | 2.9 ± 0.2 | 3.2 ± 0.3 | 0.541 |
| Twenty four hours | 2.8 ± 0.1 | 2.8 ± 0.1 | 2.9 ± 0.2 | 3.1 ± 0.2 | 0.561 |
| **Weight loss outcomes** |  |  |  |  |  |
| Weight |  |  |  |  |  |
| Six months | 91.0 ± 1.7 | 93.0 ± 2.6 | 97.8 ± 5.9 | 98.4 ± 6.5 | 0.509 |
| Year 1 | 81.2 ± 1.7 | 82.1 ± 2.3 | 85.2 ± 5.2 | 85.8 ± 5.9 | 0.834 |
| BMI |  |  |  |  |  |
| Six months | 33.1 ± 0.6 | 33.6 ± 0.8 | 35.7 ± 1.8 | 35.6 ± 2.6 | 0.338 |
| Year 1 | 29.4 ± 0.5 | 29.3 ± 0.7 | 31.1 ± 1.5 | 30.9 ± 2.2 | 0.599 |
| TWL |  |  |  |  |  |
| Six months | 24.9 ± 0.7 | 23.6 ± 0.7 | 23.8 ± 1.5 | 30.2 ± 1.7 | 0.013 |
| Year 1 | 33.6 ± 0.7 | 33.8 ± 1.0 | 33.6 ± 1.7 | 39.0 ± 2.4 | 0.210 |
| EWL |  |  |  |  |  |
| Six months | 50.7 ± 1.4 | 49.3 ± 1.9 | 45.9 ± 3.8 | 55.1 ± 5.0 | 0.341 |
| Year 1 | 68.2 ± 1.6 | 68.9 ± 2.4 | 64.4 ± 4.1 | 70.4 ± 5.0 | 0.782 |
| **Post-operative Complications** | 5 (4.9) | 4 (6.2) | 0 (0.0) | 0 (0.0) | 0.921 |
| **Types** |  |  |  |  |  |
| Bleeding | 0 (0.0) | 2 (3.1) | 0 (0.0) | 0 (0.0) | 0.347 |
| Chest Infection | 4 (3.9) | 0 (0.0) | 0 (0.0) | 0 (0.0) | 0.402 |
| Internal Hernia | 0 (0.0) | 0 (0.0) | 0 (0.0) | 0 (0.0) |  |
| Port Site Hernia | 0 (0.0) | 0 (0.0) | 0 (0.0) | 0 (0.0) |  |
| Superficial Wound Infection | 1 (1.0) | 0 (0.0) | 0 (0.0) | 0 (0.0) | 1.000 |
| UTI | 0 (0.0) | 2 (3.1) | 0 (0.0) | 0 (0.0) | 0.343 |
| Wond Infection | 0 (0.0) | 0 (0.0) | 0 (0.0) | 0 (0.0) |  |
| **Readmission** | 11 (10.8) | 2 (3.1) | 0 (0.0) | 1 (12.5) | 0.154 |
| **Causes** |  |  |  |  |  |
| Bleeding | 0 (0.0) | 2 (3.1) | 0 (0.0) | 0 (0.0) | 0.339 |
| Internal Hernia | 0 (0.0) | 0 (0.0) | 0 (0.0) | 0 (0.0) |  |
| Nausea | 5 (4.9) | 0 (0.0) | 0 (0.0) | 1 (12.5) | 0.095 |
| Port Site Hernia | 0 (0.0) | 0 (0.0) | 0 (0.0) | 0 (0.0) |  |
| Vomiting | 5 (4.9) | 1 (1.5) | 0 (0.0) | 0 (0.0) | 0.724 |

Data are presented as mean ± standard error (SE) for continuous variables and number (percentage) for categorical variables. This table reports a substance-specific analysis restricted to TST-positive patients, stratified by predominant substance exposure (tramadol only, cannabis only, sedatives ± opiates, and poly-substance), to explore heterogeneity within the TST-positive group. Poly-substance use included three patients positive for tramadol plus benzodiazepines and five positive for tramadol, opiates, and cannabis. For continuous variables, p values were obtained using the Kruskal–Wallis test. For categorical variables, Monte Carlo–simulated Fisher’s exact tests were used due to small cell counts. These analyses were conducted to assess descriptive patterns across substance categories and are intended as exploratory, given the limited sample size in some subgroups. Statistically significant results (p < 0.05) are indicated in bold italic.

| **Table S6:** Global longitudinal generalized estimating equation (GEE) analysis of postoperative VAS pain scores among TST-positive patients, comparing substance-specific exposure groups and adjusting for sex and smoking | | |
| --- | --- | --- |
| **Factor** | **MD (95% CI)** | **p** |
| **Time** |  |  |
| Baseline | Reference |  |
| 1 hour | 3.27 (3.21, 3.34) | ***< 0.001*** |
| 6 hours | 2.27 (2.21, 2.34) | ***< 0.001*** |
| 12 hours | 1.27 (1.21, 1.34) | ***< 0.001*** |
| 24 hours | 1.23 (1.17, 1.29) | ***< 0.001*** |
| **Substance** |  |  |
| Tramadol only | Reference |  |
| Cannabis only | -0.01 (-0.25, 0.23) | 0.933 |
| Sedatives ± Opiates | 0.18 (-0.18, 0.54) | 0.315 |
| Poly-substance | 0.39 (-0.01, 0.80) | 0.056 |
| **Smoking** |  |  |
| No | Reference |  |
| Yes | 0.04 (-0.21, 0.28) | 0.752 |
| **Sex** |  |  |
| Male | Reference |  |
| Female | -0.65 (-0.89, -0.41) | ***< 0.001*** |

Results are presented as mean differences (MD) with 95% confidence intervals (CI) and corresponding p values. Postoperative Visual Analog Scale (VAS) pain scores measured at baseline, 1 hour, 6 hours, 12 hours, and 24 hours were analyzed using generalized estimating equations (GEE) with an exchangeable working correlation structure to account for within-subject correlation. Time was modeled as a categorical variable with baseline as the reference category. Substance exposure (tramadol only, cannabis only, sedatives ± opiates, and poly-substance use) was included to assess heterogeneity within the TST-positive group, with tramadol-only use as the reference category. Poly-substance use included three patients positive for tramadol plus benzodiazepines and five positive for tramadol, opiates, and cannabis. Models were adjusted for sex and smoking status. This analysis is exploratory, given the limited sample size in some substance subgroups. Statistically significant results (p < 0.05) are indicated in bold italic. GEE, generalized estimating equation; VAS, visual analogue scale; MD, mean difference; CI, confidence interval; TST, toxicology screening test.

| **Table S7:** Substance-specific multivariable linear regression analyses of anesthesia requirements, recovery characteristics, and postoperative opioid use among TST-positive patients (adjusted for sex and smoking) | | | | | | |
| --- | --- | --- | --- | --- | --- | --- |
| **Factor** | **Propofol Dose (mg)** | | **Intraoperative Fentanyl Dose (mcg)** | | **Perioperative Morphine Dose (mg)** | |
|  | **MD (95% CI)** | **p** | **MD (95% CI)** | **p** | **MD (95% CI)** | **p** |
| **Substance** |  |  |  |  |  |  |
| Tramadol only | Reference |  | Reference |  | Reference |  |
| Cannabis only | 0.72 (-6.53, 7.97) | 0.845 | 0.26 (-4.69, 5.22) | 0.917 | 0.10 (-0.47, 0.66) | 0.730 |
| Sedatives ± Opiates | 9.01 (-3.20, 21.22) | 0.147 | 6.08 (-2.26, 14.43) | 0.152 | 0.58 (-0.37, 1.53) | 0.232 |
| Poly-substance | 18.37 (2.19, 34.56) | ***0.026*** | 9.60 (-1.46, 20.67) | 0.088 | 0.88 (-0.38, 2.14) | 0.168 |
| **Smoking** |  |  |  |  |  |  |
| No | Reference |  | Reference |  | Reference |  |
| Yes | -1.05 (-8.48, 6.38) | 0.781 | -0.62 (-5.70, 4.46) | 0.809 | -0.05 (-0.63, 0.53) | 0.861 |
| **Sex** |  |  |  |  |  |  |
| Male | Reference |  | Reference |  | Reference |  |
| Female | -17.87 (-24.99, -10.75) | < 0.001 | -12.70 (-17.57, -7.84) | < 0.001 | -1.68 (-2.23, -1.12) | < 0.001 |
| **Factor** | **Recovery Time (min)** | | **Recovery Status score** | | **First Opioid Request** | |
|  | **MD (95% CI)** | **p** | **MD (95% CI)** | **p** | **MD (95% CI)** | **p** |
| **Substance** |  |  |  |  |  |  |
| Tramadol only | Reference |  | Reference |  | Reference |  |
| Cannabis only | 0.01 (-1.02, 1.04) | 0.987 | 0.00 (-0.32, 0.32) | 0.996 | -2.28 (-2.45, -2.12) | ***< 0.001*** |
| Sedatives ± Opiates | 1.83 (0.10, 3.56) | ***0.039*** | -1.47 (-2.01, -0.92) | ***< 0.001*** | -0.78 (-1.06, -0.50) | ***< 0.001*** |
| Poly-substance | 2.25 (-0.04, 4.55) | 0.054 | 0.59 (-0.13, 1.31) | 0.109 | -2.07 (-2.44, -1.70) | ***< 0.001*** |
| **Smoking** |  |  |  |  |  |  |
| No | Reference |  | Reference |  | Reference |  |
| Yes | -0.01 (-1.07, 1.04) | 0.981 | -0.00 (-0.33, 0.33) | 0.985 | 0.08 (-0.09, 0.25) | 0.330 |
| **Sex** |  |  |  |  |  |  |
| Male | Reference |  | Reference |  | Reference |  |
| Female | -2.43 (-3.44, -1.42) | < 0.001 | -0.59 (-0.90, -0.27) | < 0.001 | -0.01 (-0.17, 0.15) | 0.914 |

Results are presented as mean differences (MD) with 95% confidence intervals (CI) and corresponding p values. Separate multivariable linear regression models were fitted for each outcome among TST-positive patients, comparing substance-specific exposure groups (tramadol only, cannabis only, sedatives ± opiates, and Tramadol-based poly-substance use), with tramadol-only use as the reference category. Poly-substance use included three patients positive for tramadol plus benzodiazepines and five positive for tramadol, opiates, and cannabis. All models were adjusted for sex and smoking status. These analyses were conducted to explore heterogeneity within the TST-positive group and are exploratory, given the limited sample size in some substance subgroups. Statistically significant results (p < 0.05) are indicated in bold italic. MD, mean difference; CI, confidence interval; TST, toxicology screening test.

| **Table S8:** Substance-specific global longitudinal generalized estimating equation (GEE) analysis of body mass index (BMI) changes after bariatric surgery among TST-positive patients (adjusted for sex and smoking) | | |
| --- | --- | --- |
| **Factor** | **MD (95% CI)** | **p** |
| **Time** |  |  |
| Baseline | Reference |  |
| 6 months | -11.02 (-11.50, -10.55) | ***< 0.001*** |
| 1 year | -15.21 (-15.84, -14.58) | ***< 0.001*** |
| **Substance** |  |  |
| Tramadol only | Reference |  |
| Cannabis only | 0.70 (-1.15, 2.56) | 0.458 |
| Sedatives ± Opiates | 2.05 (-1.29, 5.38) | 0.229 |
| Poly-substance | 3.07 (-1.46, 7.61) | 0.184 |
| **Smoking** |  |  |
| No | Reference |  |
| Yes | -2.65 (-4.39, -0.91) | ***0.003*** |
| **Sex** |  |  |
| Male | Reference |  |
| Female | -0.92 (-2.79, 0.95) | 0.336 |

Results are presented as mean differences (MD) with 95% confidence intervals (CI) and corresponding p values. Body mass index (BMI) measured preoperatively, at 6 months, and at 1 year after bariatric surgery was analyzed using generalized estimating equations (GEE) with an exchangeable working correlation structure to account for within-subject correlation. Time was modeled as a categorical variable with baseline BMI as the reference category. Substance exposure (tramadol only, cannabis only, sedatives ± opiates, and poly-substance use) was included to explore heterogeneity within the TST-positive group, with tramadol-only use as the reference category. Poly-substance use included three patients positive for tramadol plus benzodiazepines and five positive for tramadol, opiates, and cannabis. Models were adjusted for sex and smoking status. This analysis is exploratory, given the limited sample size in some substance subgroups. Statistically significant results (p < 0.05) are indicated in bold italic. GEE, generalized estimating equation; BMI, body mass index; MD, mean difference; CI, confidence interval; TST, toxicology screening test.
